# Quinacrine induces apoptosis in cancer cells by forming a functional bridge between TRAIL-DR5 complex and modulating the mitochondrial intrinsic cascade

**DOI:** 10.18632/oncotarget.11335

**Published:** 2016-08-17

**Authors:** Sarita Das, Neha Tripathi, Ranjan Preet, Sumit Siddharth, Anmada Nayak, Prasad V. Bharatam, Chanakya Nath Kundu

**Affiliations:** ^1^ Cancer Biology Division, KIIT School of Biotechnology, KIIT University, Patia, Bhubaneswar, Odisha, 751024, India; ^2^ National Institute of Pharmaceutical Education and Research (NIPER), SAS Nagar, Mohali, Punjab, 160062, India

**Keywords:** quinacrine (QC), tumor necrosis factor related apoptosis inducing ligand (TRAIL), death receptor (DR5), breast cancer, apoptosis

## Abstract

Death Receptor 5 (DR5) is known to be an important anti-cancer drug target. TRAIL is a natural ligand of DR5, but its drug action is limited because of several factors. A few agonistic ligands were identified as TRAIL-DR5 axis modulators, which enhance the cellular apoptosis. Literature suggest that quinacrine (QC) acts as a DR5 agonistic ligand. However, the detailed mechanism explaining how QC interacts with TRAIL-DR5 axis has not been established. Also focused *in vitro* and *in vivo* experimental analysis to validate the hypothesis is not yet performed. In this work, extensive studies have been carried out using *in silico* analysis (molecular dynamics), *in vitro* analysis (cell based assays) and *in vivo* analysis (based on mice xenograft model), to delineate the mechanism of QC action in modulating the TRAIL-DR5 signaling. The MD simulations helped in identifying the important residues contributing to the formation of a QC-TRAIL-DR5 complex, which provide extra stability to it, consequently leading to the enhanced cellular apoptosis. QC caused a dose dependent increase of DR5 expression in cancer cells but not in normal breast epithelial cells, MCF-10A. QC showed a synergistic effect with TRAIL in causing cancer cell apoptosis. In DR5-KD MCF-10A-Tr (DR5 knocked down) cells, TRAIL+ QC failed to significantly increase the apoptosis but over expression of full length DR5 in DR5-silence cells induced apoptosis, further supporting DR5 as a drug target for QC. An increase in the release of reactive species (ROS and RNS) and activation of enzymes (FADD, CASPASES 3, 8, 9 and cytochrome-C) indicated the involvement of mitochondrial intrinsic pathway in TRAIL+QC mediated apoptosis. *In vivo* study pointed out that TRAIL+QC co-administration increases the expression of DR5 and reduce the tumor size in xenograft mice. This combined *in silico*, *in vitro* and *in vivo* analysis revealed that QC enhances the cellular apoptosis via the modulation of TRAIL-DR5 complexation and the mitochondrial intrinsic pathway.

## INTRODUCTION

Tumor Necrosis factor Related Apoptosis Inducing Ligand (TRAIL), also known as Apo-2L, is a cytokine of TNF (Tumor Necrosis Factor) group. The anti-cancer potential of this biopharmaceutical agent has been highlighted, especially due to its ability to specifically kill cancer cells without affecting the normal cells [1]. TRAIL causes cancer cell death by inducing apoptosis via death receptor (DR) signaling cascade. Two functional death domain containing receptors and three decoy receptors have been found to interact with TRAIL. The cellular apoptosis is a result of the interaction between TRAIL and functional death domain containing receptors *i.e.* DR4 (TRAIL-R1) and DR5 (TRAIL-R2/Killer) [1, 2]. The decoy receptors *i.e.* DCR1 (TRAIL-R3), DCR2 (TRAIL-R4) and osteoprotegrin (opg), do not have functional death domain and hence play a key role in inhibiting apoptosis by interacting with TRAIL.

Cellular apoptosis induced on TRAIL binding to DR4/DR5 is a multistep process, involving receptor trimerization, formation of Death Inducing Signaling Complex (DISC) and subsequent cell death. DISC recruits Fas-Associated protein with Death Domain (FADD) and this leads to the activation of pro-caspase 8 to CASPASE 8 *via* autocatalysis. CASPASE 8 then induces apoptosis via two different cascades *i.e.* extrinsic and intrinsic pathways [1]. Intrinsic pathway involves cleavage of Bcl-2 homology domain 3 (BH3) interacting-domain death agonist (Bid) to form truncated Bid (tBid), which in turn interacts with the pro-apoptotic B-cell lymphoma 2 (Bcl2) family members Bcl-2-associated X protein(BAX) and BAK (Bcl-2-like protein 4). This interaction stimulates the release of cytochrome C (Cyt C) from the mitochondria, formation of apoptosome, recruitment of CASPASE 9 and activation of CASPASE 3 in a sequential manner, ultimately resulting into cellular apoptosis.

Recent research efforts were focused on DR5 as a therapeutic target; several antibodies under clinical studies, were developed to specifically target DR5 but not DR4. The reasons for such choice can be listed as given below: i) DR5 is expressed in higher concentration on the surface of tumor cells than DR4 [3]; ii) DR5 is more potent than DR4 in causing apoptosis [4]; iii) DR5 is reported to have higher affinity for TRAIL than DR4 at physiological temperatures [5, 6]; iv) frequent mutations of DR4 gene are observed in cancer patients [7]; v) DR4 can function by binding to both cross-linked and non-cross-linked TRAIL but DR5 signals only *via* cross-linked TRAIL [8]; vi) TRAIL-DR5 complex is reported to be the most organized complex that can serve as an ideal model for the development of DR5 agonistic antibodies [9]; vii) mice models are considered as ideal for *in vivo* studies because in mice, only DR5 receptor is expressed [10]; viii) the DR4 activity is p53 dependent and p53 mutations are very frequent in the cancer patients [11]. The p53 independency of DR5 adds another reason for DR5 being the preferred anti-cancer drug target.

TRAIL is recognized as a potent agent for the treatment of cancer [12, 13]. The limiting factors for its usage are development of resistance for TRAIL due to (i) its repeated exposure [14], (ii) interaction of TRAIL with its decoy receptors (DCR1, DCR2 and opg), (iii) mutational deletion of its functional death receptors DR4 and DR5, (iv) over expression of anti-apoptotic markers (BCL2 family proteins), Inhibitor of apoptosis protein (IAP) like survivin, cellular inhibitor of apoptosis protein (CIAP) and cellular FLICE(FADD-like IL-1β-converting enzyme) like inhibitory protein (C-FLIP) an inhibitor of the DISC formation [15] and (v) impaired oligomerization of DR5 on the cell surface [2]. Combination therapy is often adopted as an alternative treatment policy to enhance the efficacy of TRAIL [16]. There are several reports, pointing towards the enhanced therapeutic potency of TRAIL in combination with curcumin [17], mangostin-alpha [2], resveratrol [18], cisplatin [19], doxorubicin [20] and several other drugs. One of the genuine problems in the existing combination therapy is that most of the agents work through p53 dependent pathways [21]. Several DR5 independent pathways such as C-Jun N-terminal kinase (JNK), mitogen activated protein kinase (MAPK), extracellular signal-regulated protein kinase (ERK), nuclear factor kappa B (NF-kβ) and Janus Kinase/signal transducers and activators of transcription (JAK/STAT) pathways are also capable of transmitting apoptotic signals [22–24].

Quinacrine (QC) is a well-known, age old, anti-malarial drug and is recently re-established as an anti-cancer drug [25–28]. Reports on the anti-cancer efficacy of QC on cancer cell lines suggests that it causes apoptosis by arresting the cell cycle in the S-phase via inhibition of the topoisomerase activity, induction of p53 and p21 [29, 30] and inhibition of NF-kβ and Wnt-TCF signaling through adenomatous polyposis coli (APC) gene [31]. Recently, our group established that the nanoparticles of QC exhibit anti-cancer and anti-angiogenic effects. It is specifically significant because these hybrid nanoparticles of silver and QC showed efficacy on cancer stem cells (CSCs) [32]. The higher selectivity of QC for cancer cells further offers advantages in terms of toxicity against normal cells [29]. Eferl *et al.* reported that bioactive QC stabilizes p53 by blocking p53 ubiquitination and therefore, rescues p53 from proteosomal degradation [21]. In limited experiments, Wang *et al.* [11] and Jani *et al.* [33] observed that the enhanced cellular apoptosis is due to synergistic effect of QC and TRAIL. In spite of these clues, the detailed mechanism of action elaborating the interaction between TRAIL, DR5 and QC at atomic level is not known.

In this work, synergistic effect of QC and TRAIL on cellular apoptosis enhancement is demonstrated in breast and kidney cancer cell lines as well as in cigarette smoke condensate induced-cancer cell lines. A systematic evaluation of the mechanism for the cell death caused by the co-administration of QC and TRAIL via DR5 regulation is undertaken. Molecular modeling techniques are adopted to analyze the structural aspects of TRAIL-DR5 binary complex, Fab-TRAIL-DR5 ternary complex and QC-TRAIL-DR5 ternary complex. The molecular docking studies were performed to identify the binding site of QC. The various complexes used in the study (TRAIL-DR5, Fab-TRAIL-DR5 and QC-TRAIL-DR5) were subjected to molecular dynamics simulations and binding energy calculations. The *in silico* and *in vitro* DR5 mutational and *in vivo* experiments were used to further validate the obtained data. The combined results helped in postulating the mechanism underlying the enhanced cellular apoptosis exhibited by QC.

## RESULTS

### Structural origin of the enhanced TRAIL-DR5 binding due to QC

Jani et al. [33] suggested the synergistic effect of QC and TRAIL in causing cellular apoptosis. This was further supported from the observations by Wang et al. [11], who reported that QC sensitizes hepatocellular carcinoma cells to TRAIL. These observations may be rationalized in terms of structural factors. Considering that TRAIL directly binds to DR5, QC may be enhancing this binding by improving the interactions between these two. To evaluate this hypothesis, molecular docking and molecular dynamics studies have been taken up. The co-ordinates used for the molecular modeling studies are taken from the PDB ID 4N90 (which is a complex containing three molecules each of DR5, TRAIL and Fab fragment of AMG 655). The entire TRAIL-DR5-Fab assembly is an A3 arrangement where each A corresponds to TRAIL-DR5-Fab complex. Fab is the antigen binding domain of AMG 655 antibody. In order to understand the structural properties of TRAIL-DR5 complex and identify the representative functional unit of the TRAIL-DR5 hexameric complex, molecular modeling studies were undertaken. Three molecules of DR5 binds to the trimer of TRAIL such that each DR5 (chain R in 4N90) occupies the groove formed between two TRAIL molecules (chain A and chain C in 4N90), thus interacting with both the TRAIL molecules. The molecular modeling studies revealed that Fab (chain E and chain D) affects the complexation DR5 with TRAIL belonging to chain A (see supporting information for details). Thus, TRAIL-DR5 (*i.e.* chain A and chain R in 4N90) can be considered as the representative model system for studying the effect of DR5 agonistic ligands on TRAIL-DR5 assembly. Therefore, in the molecular modeling work discussed below, a binary complex of TRAIL and DR5 is considered. The chains (from 4N90) used for the molecular modeling studies are chain R (DR5), chain A (TRAIL), chain E (first chain of Fab) and chain D (second chain of Fab).

Binding pocket identification for the QC in the DR5 is a pre-requisite to proceed. With an assumed mechanistic analogy with the death receptor agonistic antibody AMG 655 [34], QC is expected to bind at the region where Fab of AMG 655 binds. Further, the calculation of vertical ionization energy (Table S1), p*K*a (Figure S1G) and analysis of various ionic species concentrations at pH range 0 to 14 (Figure S1 and Table S2) showed that the QC has a higher propensity to exist in *N1,N3*-diprotonated form (Figure S1D). QC^2+^ is expected to occupy a region around the negatively charged (acidic) residues, such as Asp and Glu, at the interface of Fab, TRAIL and DR5. Therefore, molecular docking of diprotonated QC was performed at the centroid of the DR5 and TRAIL residues (ThrA129, ArgA130, SerA145, MetA260, ArgB65, CysB66, GluB70, ValB71, GluB72 and GluB87) interacting with the Fab in the crystal structure. Subsequently, molecular dynamics simulation was performed for the best docked pose of QC in TRAIL-DR5 complex.

**Figure 1:**
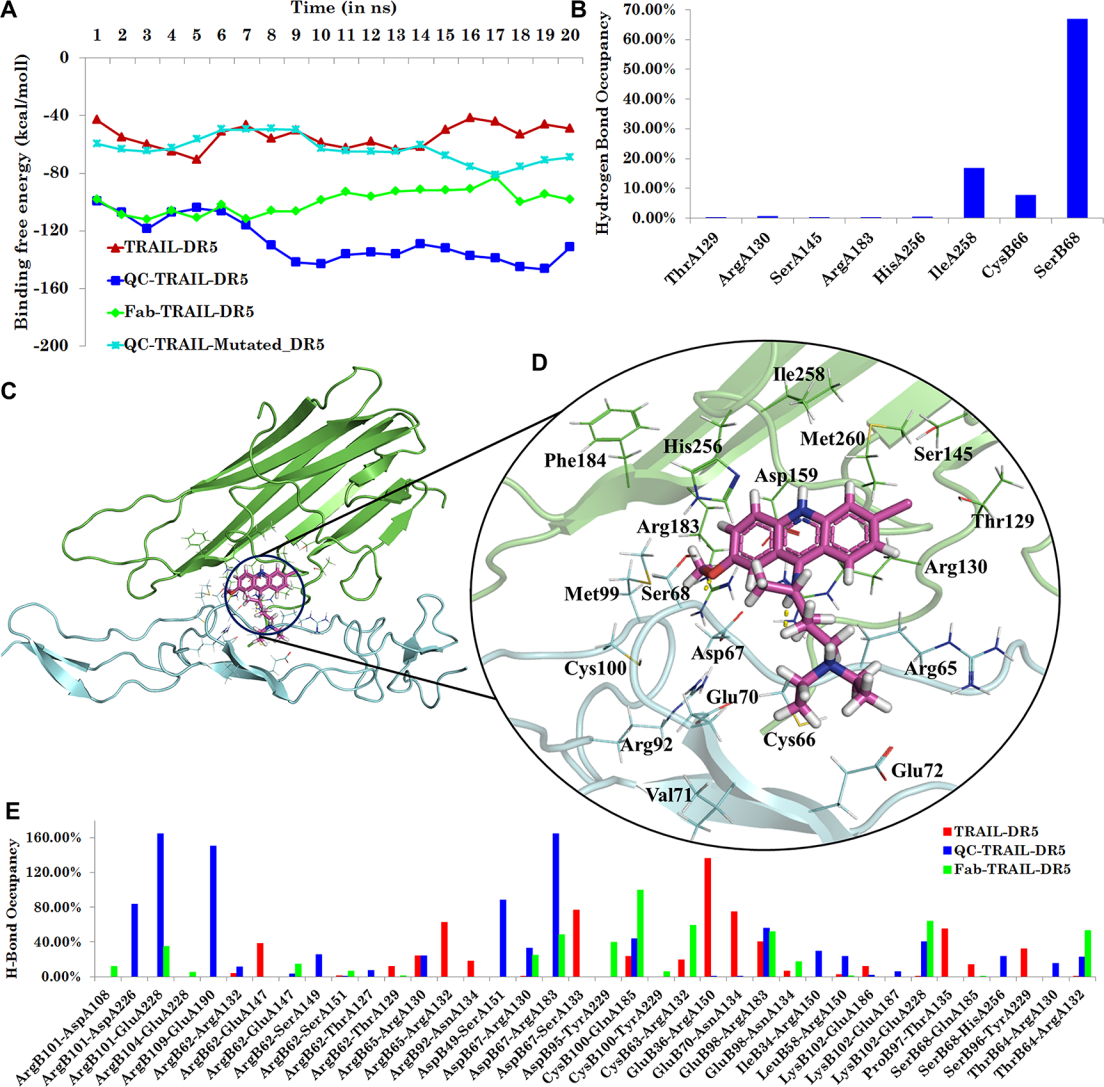
Analysis of molecular dynamics simulation for various complexes (**A**) Binding energy for various systems *i.e.* binary complex TRAIL-DR5, ternary complexes Fab-TRAIL-DR5, QC-TRAIL-DR5 and QC-TRAIL-Mutated-DR5. (**B**) Hydrogen bonds occupancy for the QC interaction with residues of TRAIL and DR5 over the last ns trajectory in the ternary complex QC-TRAIL-DR5. (**C**) QC binds near the TRAIL-DR5 interface. (**D**) The molecular recognition interactions of QC in TRAIL-DR5 complex. (**E**) Hydrogen bond occupancy analysis between DR5 residues and TRAIL residues (x-axis) in the three complexes.

Molecular dynamics analysis was performed on the complexes TRAIL-DR5, Fab-TRAIL-DR5 and QC-TRAIL-DR5. The data for the analysis is presented in Figure 1. All the systems were stabilized after 10 ns (Figure S5). There were no major changes noticed in the secondary structure of the macromolecules, during molecular dynamics simulation (Table S3 and Figure S6). The calculated binding energy (Figure 1A) also confirmed that the systems showed minimal fluctuations after 10 ns simulation run. The binding energy between TRAIL and DR5 averages to −53.69 ± 10.73 kcal/mol, whereas that between TRAIL, DR5 and Fab averages to −91.50 ± 10.93 kcal/mol. This indicates that Fab significantly stabilized the TRAIL and DR5 complexation.

QC could be comfortably docked near the TRAIL-DR5 interface and this ternary complex (Figure 1C) was stable during the course of molecular dynamics simulations. The analysis of hydrogen bond occupancies between QC and the macromolecules over the last ns (20th) trajectory showed stable hydrogen bonding interaction with IleA258, CysB66 and SerB68 (Figure 1B). Several intermittent hydrogen bonds such as with ThrA129, ArgA130, SerA145, ArgA183 and HisA256, were also observed between QC and TRAIL. The system was stabilized during the period of MD simulation run, as indicated by the RMSD analysis, (Figure S5). On an average, around two hydrogen bonds were observed during the molecular dynamics simulation (Figure S7B). The other residues contributing to the binding of QC in TRAIL-DR5 complex were AspA159, ArgB65, AspB67 and GluB70 (Figure 1D). The ternary complex between QC-TRAIL-DR5, generated from molecular docking studies was used to understand the effect of QC binding on TRAIL-DR5 interactions. The binding energy between the TRAIL and DR5 in the ternary complex QC-TRAIL-DR5 for each ns is plotted in Figure 1A. The average binding energy for QC-TRAIL-DR5 complexation was calculated to be −137.50 ± 10.75 kcal/mol. This value is much higher than that in the binary complex, TRAIL-DR5 as well as the ternary complex Fab-TRAIL-DR5. These results indicate that QC efficiently binds between the TRAIL and DR5, increasing the complexation between these two macromolecules. The increase in the binding energy is mainly due to an increase in the hydrogen bonding contacts and the van der Waals contributions between TRAIL and DR5, as shown in Figure 1E and Figure S7A. Further, the volume of interfacial cavities was significantly reduced in QC-TRAIL-DR5 complex (Figure S8), bringing the TRAIL and DR5 closer. In contrast, when the effect of QC binding on TRAIL-DR4 complexation was analysed, no significant difference was observed between the TRAIL-DR4 complex and QC-TRAIL-DR4 complex, indicating the possible DR5 specificity of QC (Figure S9).

In order to understand the importance of DR5 residues CysB66 and SerB68 in mediating the death promoting action of QC, *in silico* mutational studies were performed. The mutations Cys66Gly and Ser68Ala were inserted in the DR5 of ternary complex QC-TRAIL-DR5. The complex was subjected to molecular dynamics simulation for 20 ns (using the similar conditions as that of other three complexes). The Figure 1A (cyan) shows the fluctuations in the binding energy of TRAIL and DR5 in the ternary complex QC-TRAIL-Mutated-DR5 which is well within the range similar to that of the TRAIL-DR5 binary complex (Figure 1A, red). The mutation of these two residues led to the failure of QC to cause any significant increase in the binding energy of TRAIL and DR5. Thus, the *in silico* studies strongly suggest that QC enhances the TRAIL-DR5 interaction. To confirm the observations made from *in silico* studies, *in vitro* studies were performed.

### QC-mediated apoptosis in breast cancer cells is DR5 dependent

**Figure 2:**
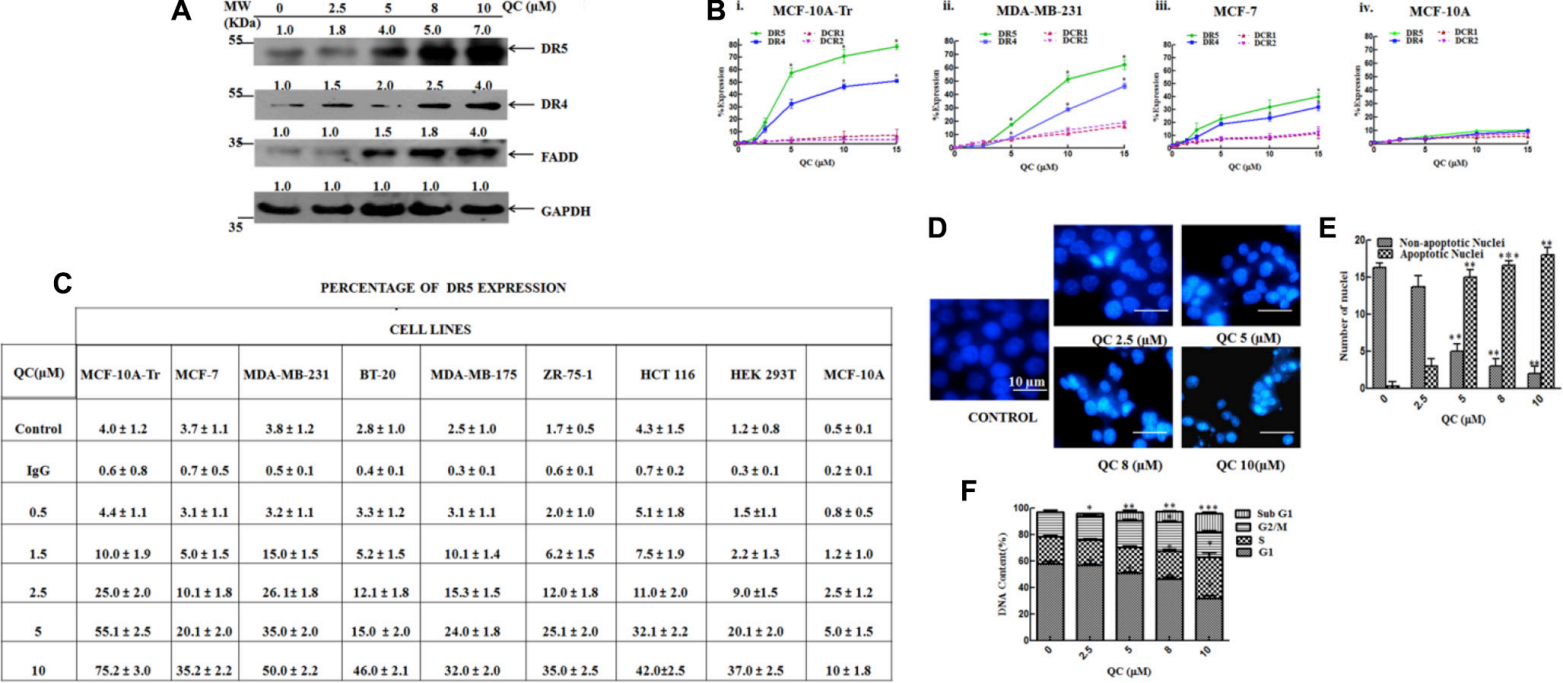
QC caused apoptosis in cancer cells by increasing the expressions of death receptors (**A**) Expressions of proteins in QC treated MCF-10A-Tr cells. (**B**) Expressions of different receptors in cell surface measured by FACS. i) MCF-10A-Tr, ii) MDA-MB-231, iii) MCF-7, and iv) MCF-10A cells. (**C**) Expression of DR5 in different cells after QC exposure. (**D**) Apoptosis measured by DAPI staining. (**E**) Graph representing the number of apoptotic and non-apoptotic nuclei in Figure 2D. (**F**) Measurement of apoptosis by FACS analysis of MCF-10A-Tr cells treated with QC. The images shown are representative of three different experiments. Data are the mean ± SD of three different experiments.

The molecular modeling studies provided a possible hint for the DR5 mediated apoptosis enhancement by QC. To further validate these speculations, expressions of the death receptors (DR4 and DR5) in breast cancer cells after exposure to QC were evaluated. For this purpose, the expressions of DR4 and DR5 were measured after exposure to QC in different breast cancer cells including cigarette smoke induced breast cancer (MCF-10A-Tr) cells. Figure 2A demonstrates the dose dependent increase of DR5, DR4 and adaptor protein FADD expressions with increasing dose of QC in MCF-10A-Tr cells. Approximately, 7 fold and 4 fold increase in the expressions of DR5 and DR4 were noted in comparison to control. More than 4 fold increase in the expression of FADD was noted in cells treated with 10 μM QC. In order to evaluate whether QC can affect the expressions of death receptors other than DR4 and DR5, we have taken multiple breast cancer cells (MCF-10A-Tr, MDA-MB-231, MCF-7, BT-20, MDA-MB-175, and ZR-75-1), kidney cancer (HEK-293T), and colon cancer cells (HCT-116) along with normal breast cells MCF-10A and treated with different concentrations of QC. As most of the death receptors are expressed on the cell surface, the expressions of the proteins was measured by fluorescence associated cell sorting (FACS) analysis, after incubating the cells with specific antibody against the receptors. The expressions of DR4 and DR5 were significantly increased in tested cell lines (Figure 2B), with no significant change in the expressions of DCR1 and DCR2. Figure 2C shows the relative expression of DR5 in different cancer cell lines along with MCF-10A. It was also noticed that the DR5 expression was much higher in MCF-10A-Tr (75%) cells than MDA-MB-231 cells (50%) and MCF-7 cells (40%) at 10 μM QC (Figure 2B.i, 2B.ii, 2B.iii and Figure 2C). Interestingly, there was no significant change in expression (approx. 10%) of any receptor was noticed in MCF-10A cells (Figure 2B.iv and Figure 2C) after QC treatment. It was also noticed that the expression of DR5 was more in comparison to DR4 in the tested cancer cell lines (Figure 2B). QC also increased the expression of DR5 in other cancer cells such as colon (HCT-116) and kidney (HEK-293T) (Figure 2C). Approximately, 38% expression of DR5 was noticed at 10 μM of QC in HCT-116 and HEK-293T cells.

To evaluate the apoptotic effect of QC in MCF-10A-Tr cells, two separate assays were performed after treating the cells with QC. First, QC treated cells were stained with 4′,6-diamidino-2-phenylindole (DAPI) nuclear staining dye and measured the shrunken, bubble-shaped and fragmented nuclei under microscope and secondly, the cells were sorted using flow cytometer after propidium iodide (PI) staining. It was noticed that apoptotic (fragmented, bubble shaped, shrunken) nuclei were increased with increasing concentrations of QC in DAPI stained cells (Figure 2D). A quantitative representation of the number of apoptotic and non-apoptotic nuclei is provided in Figure 2E. In agreement with DAPI staining data the increased number of Sub-G_1_ population (15%) was observed with increase in the dose of QC, which further proved that QC caused apoptosis in the transformed breast cancer cells (Figure 2F). We have also measured the apoptotic effect of QC in other two breast cancer cells (MCF-7 and MDA-MBA-231) using FACS analysis of PI stained cells. QC caused maximum of 14% cell death at 15 μM in MCF-7 cells and 16% cell death at 20 μM in MDA-MB-231 cells (Figure S11).

Taken together this figure indicates that QC specifically increased the expressions of death receptor protein DR4 and DR5, resulting in increased apoptosis of breast cancer cells.

### TRAIL enhances the QC mediated apoptosis in MCF-10A-Tr cells

**Figure 3:**
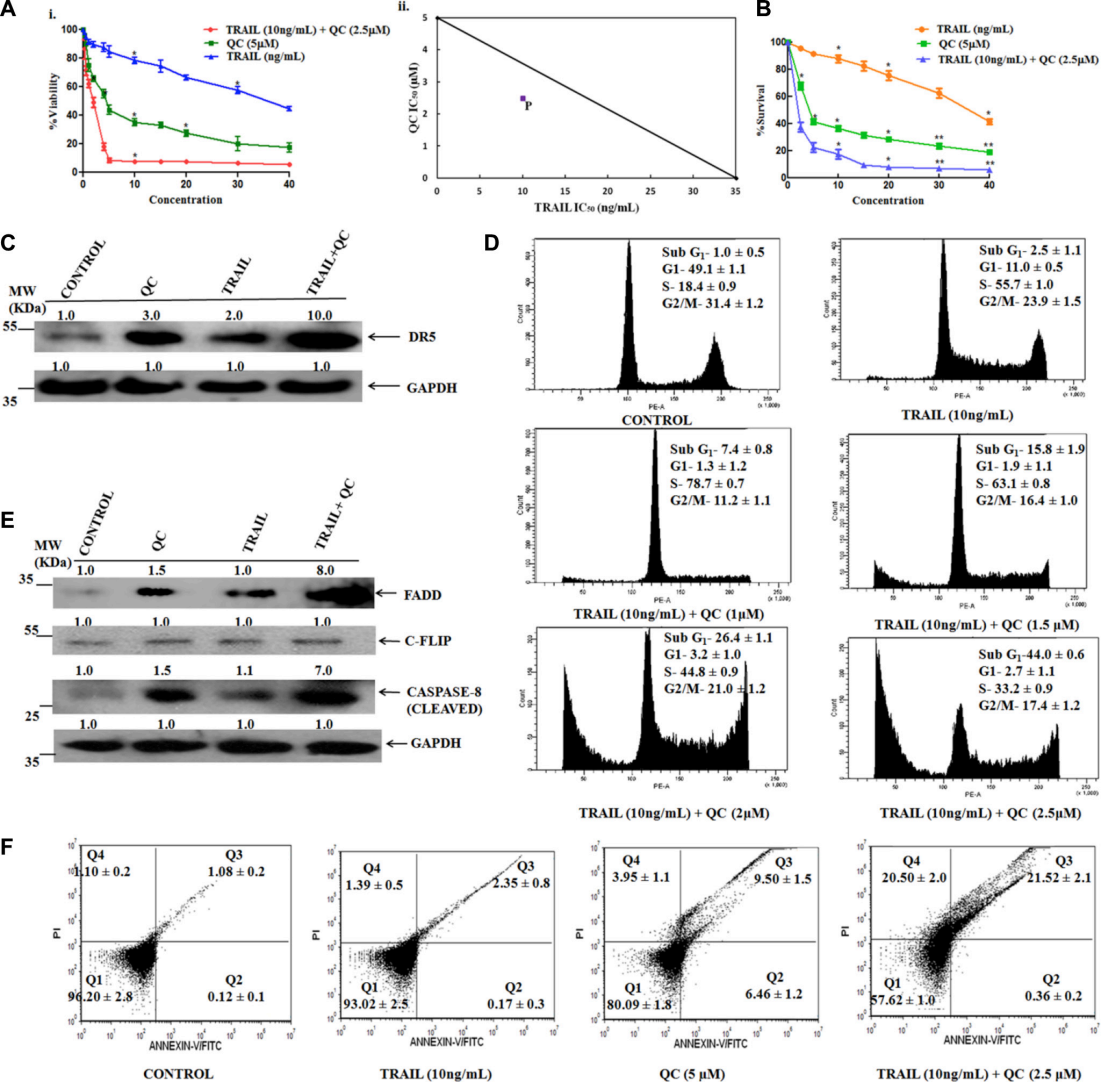
TRAIL enhances QC action for causing apoptosis in MCF-10A-Tr cells Cells were treated as mentioned in methods material section and experiments were carried out. (**A**) i) MTT cell viability assay.ii)Isobologram depicting synergistic activity of QC and TRAIL. The line showed the isobologram of QC and TRAIL and the point ‘P’ represent the 50% cell death in combined drug treatment (**B**) Clonogenic cell survival assay (**C**) Change in expression of DR5 by western blotting. (**D**) Measurement of apoptosis by FACS analysis. (**E**) Change in expression of proteins involved in DISC. (**F**) Measurement of apoptosis by annexin V-FITC/PI dual staining by using FACS analysis. Images shown are the representative of three different experiments. Data are the mean ± SD of three independent experiments.

It is well documented fact that TRAIL increased the apoptosis in cancer cells by interacting with its death receptors [2]. From the above experiments, it was noted that QC also caused apoptosis in breast cancer cells including our newly generated MCF-10A-Tr cells, by over expressing the death receptor DR5. It was noticed that although QC increased the DR5 expression by more than 75% in MCF-10A-Tr cells, but the resulting apoptosis was not more than 15% (Figure 2E). From *in silico* studies, it was speculated that QC strongly binds with TRAIL-DR5 complex and stabilizes the TRAIL-DR5 interaction. To validate the *in silico* results and to check whether TRAIL can potentiate QC and induce cell death in MCF-10A-Tr cells, a series of experiments were carried out using various concentrations of QC in TRAIL pre-exposed cells. Figure 3A demonstrates the dose dependent decrease of cell survival on treatment with QC in TRAIL-untreated cells and TRAIL pre-treated cells. QC alone can cause 50% cell death at 5 μM but similar amount of cell death occurred at 2.5 μM, when combined with TRAIL (10 ng/mL, 3 h) (Figure 3A.i). Interestingly, no appreciable cell death was noticed till 10 ng/mL of TRAIL treatment alone (Figure 3A.i). TRAIL caused 50% cell death at 35 ng/mL exposure. Figure 3A.ii represents an isobologram drawn from the IC_50_ values of TRAIL and QC obtained from the MTT assay of Figure 3A.i. It depicts the synergistic activity of QC that is well marked from the point (P) that falls below the line of additivity. A clonogenic cell survival assay was also performed to further confirm the data obtained from the MTT assay and we found similar results (Figure 3B). To further confirm the role of TRAIL in QC mediated cell death, the expression of DR5 was measured after a 48 h treatment with QC, TRAIL and TRAIL+QC. A relatively higher protein expression of DR5 (10 fold) was noticed in TRAIL+QC treated cells, as compared to QC (3 fold) and TRAIL (2 fold) treated cells (untreated cells used as reference) (Figure 3C). To correlate the increased DR5 expression with apoptosis, we have analyzed the cells by FACS after PI staining. The percent increase in Sub-G_1_ population was more in cells treated with TRAIL+QC rather than cells treated either with QC or TRAIL, individually. 44% Sub-G_1_ population cells were noted at 2.5 μM QC when combined with 10 ng/mL TRAIL (Figure 3D). The analysis of various protein components of DISC showed an increased level of FADD (8 fold) and CASPASE 8 (7 fold) and a decreased expression of C-FLIP in TRAIL+QC treated cells in comparison to untreated cells (Figure 3E).

To further support the induction of apoptosis by QC in TRAIL pre-exposed cells, an annexin V–FITC/PI dual staining assay was carried out using FACS analysis. The percentage of apoptosis (Q3) and necrosis (Q4) was significantly more in TRAIL+QC treated cells as compared to their individual treatment. Approximately, 42% apoptotic cells were noted in cells treated with TRAIL+QC combination (Figure 3F). Thus, taken together, the data shows that TRAIL increased the apoptotic effect of QC by modulating DISC complex. QC and TRAIL binds to the DR5 and caused apoptosis which is again, in correlation with the *in silico* data.

### QC caused apoptosis in MCF-10A-Tr cells by modulating the mitochondrial intrinsic cascade

**Figure 4:**
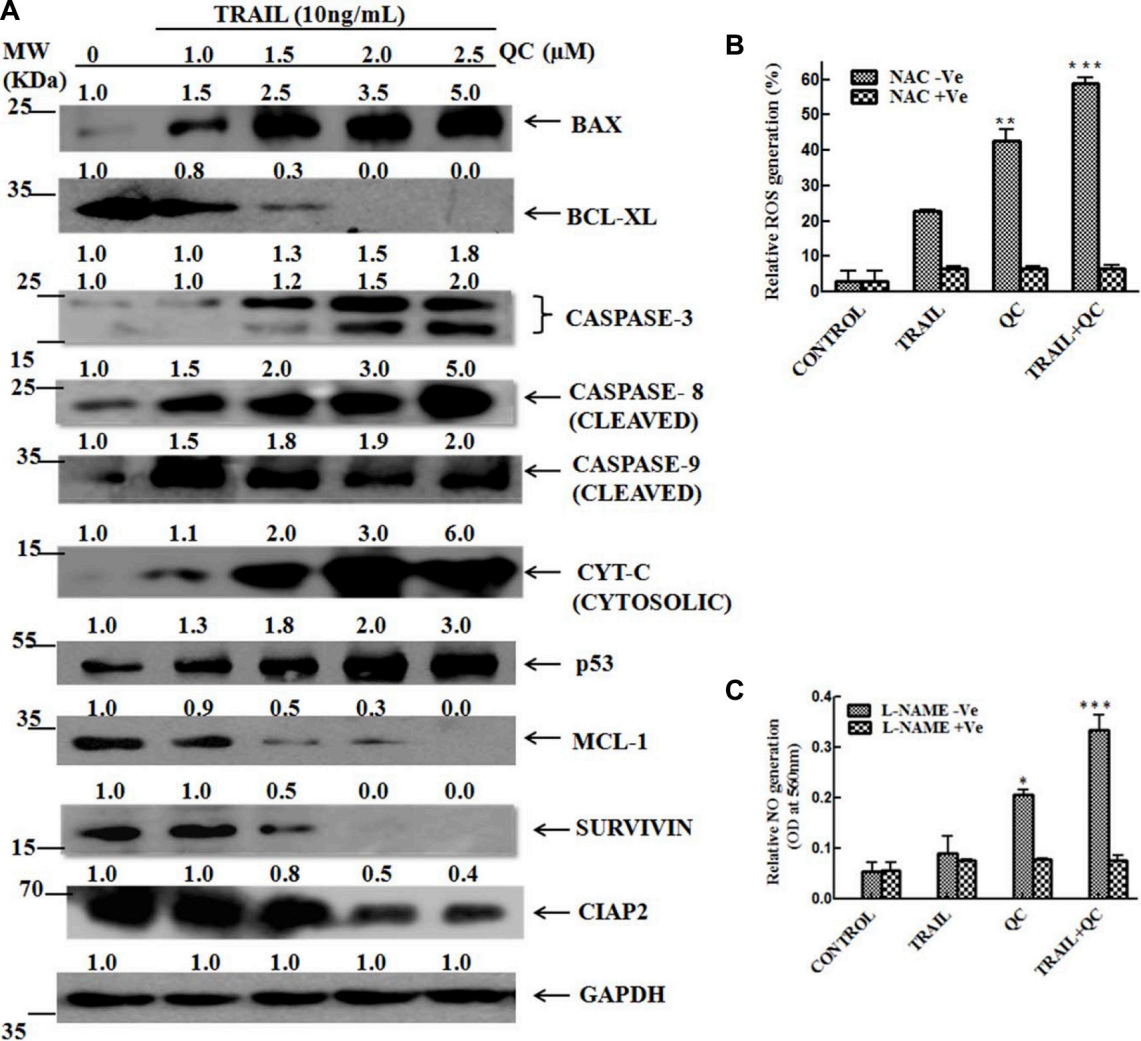
TRAIL enhances the QC mediated apoptosis in MCF-10A-Tr cells by modulation of mitochondrial intrinsic cascade Different concentrations of QC were treated in TRAIL (10 ng/mL) pre-exposed cells and experiments were carried out according to protocol motioned in methods and materials. (**A**) Expressions of proteins involved in the intrinsic cascade of apoptosis. (**B**) Measurement of ROS in presence and absence of NAC. (**C**) Production of NO in presence and absence of L-NAME. The images shown are representative of three different experiments. Data are the mean ± SD of three different experiments.

To understand the role of QC in regulation of mitochondrial intrinsic cascade, TRAIL pre-treated cells were exposed to QC and the expressions of protein components of the intrinsic signaling pathway was measured. A dose dependent CASPASE 8 activation was noted after QC treatment. Increased CASPASE 8 is one of the determinants of cells to undergo apoptosis by intrinsic pathway. A 5 fold increase in the expression of BAX and 10 fold decrease of BCL-XL was noted after QC treatment. Dose dependent increase in cleaved product of CASPASE 3, 8, 9 indicates the involvement of CASPASE (s) in causing apoptosis. More than 6 fold increase of CYTOCHROME C, a marker of intrinsic pathway of apoptosis and an indicator of mitochondrial damage, suggests that QC mediated apoptosis in MCF-10A-Tr cells is through activation of intrinsic cascade. Previously, Wang et al. reported that QC and TRAIL combination caused apoptosis in hepatocellular carcinoma cells by p53 dependent and independent pathways and decreased the expressions of MCL-1 [11]. We have also measured the status of tumor suppressor protein p53 and anti-apoptotic protein MCL-1 after QC exposure. Dose dependent increase of p53 and decrease of MCL-1 was noted in TRAIL pre-exposed QC treated cells (Figure 4A). Approximately, 3 fold p53 inductions and 10 fold MCL-1 reduction were noted after 10 μM QC exposure. Cell survival protein SURVIVIN and CIAP2 (an IAP family member) also decreased dose dependently after QC treatment (Figure 4A).

To further confirm that the TRAIL and QC mediated apoptosis in MCF-10A-Tr cells is through the damage of mitochondria, we have measured the amount of reactive oxygen species (ROS) and reactive nitrogenous species (RNS) production after treatment with TRAIL, QC and their combination (Figure 4B and Figure 4C). TRAIL and QC both increased the ROS production but the amount of ROS production was more in presence of QC (8 fold in comparison to control) than TRAIL (4 fold in comparison to control). Surprisingly, more than 12 fold increased ROS was noted when 2.5 μM of QC was added to TRAIL (10 ng/mL) pre-treated cells. To confirm that the ROS production is due to mitochondrial damage, we have pre-treated the cells with specific ROS inhibitor, N-acetyl-L-cysteine (NAC) (30 μM, 3 h) and then performed the combination treatment as above. Interestingly, no significant alteration of ROS production was noted in NAC pre-treated cells on QC treatment (Figure 4B).

RNS production was also measured after treatment with TRAIL, QC and their combination. The RNS production was increased in the presence of TRAIL, QC and their combination. This increase was highest in the combination treatment, as compared to the individual treatment. More than 4 fold induction of Nitric Oxide (NO) was noted in combination treatment as compared to untreated cells. Interestingly, pre-exposure to Nitro L-arginine methyl ester (L-NAME) (50 μM, 3 h), a specific inhibitor of RNS, significantly inhibited the production of RNS caused by combination treatment (Figure 4C). Taken together, this data suggests that TRAIL and QC caused apoptosis by modulating the mitochondrial intrinsic pathway.

### Silencing of DR5 leads to reduced apoptosis in MCF-10A-Tr cells

**Figure 5:**
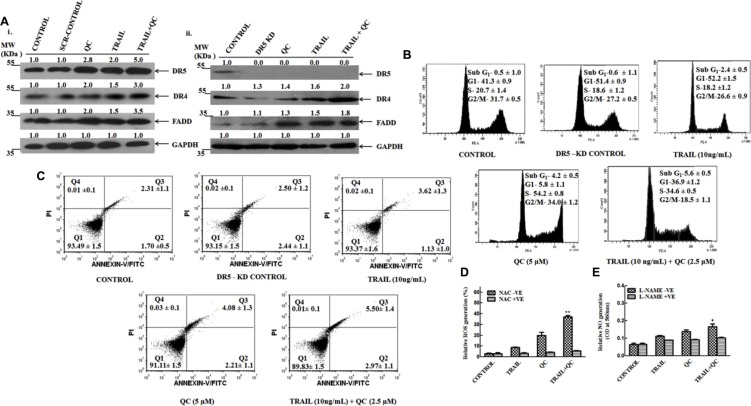
TRAIL, QC and their combination treatment reduced the apoptosis in DR5 silenced MCF-10A-Tr cells Cells were treated as mentioned in methods material section and carried out the experiments. (**A**) i) Measurement of expression of DR5, DR4 and FADD by western blotting in scramble (SCR) siRNA (h) transfected cells.ii) Measurement of expression of DR5,DR4 and FADD by western blotting in DR5 Si RNA (h) treated cells. (**B**) Measurement of apoptosis by FACS analysis in DR5 silenced cells after the drug treatment. (**C**) Determination of apoptosis by annexin V-FITC/PI dual staining in DR5 silenced cells after the drug treatment. (**D**) Production of ROS in presence and absence of NAC in DR5 silenced cells. (**E**) Production of NO in presence and absence of L-NAME in DR5 knockout cells. Images shown were representative of three different experiments. Data are the mean ± SD of three independent experiments.

In order to confirm that TRAIL and QC induced apoptosis in MCF-10A-Tr cells is mediated through DR5; the DR5 gene was silenced with the help of siRNA (h). Figure 5A.i shows the change in expressions of DR5, DR4 and FADD on TRAIL+QC treatment in scramble siRNA (SCR siRNA) transfected MCF-10A-Tr cells. There were increased expressions of these proteins observed in QC, TRAIL and their combination treated cells. Interestingly, it was noted that the increase in the protein levels were similar to the parental MCF-10A-Tr cells under same treatment conditions (Figure 2C and 2E). The Figure 5A.ii showed a complete abolition of DR5 expression after siRNA treatment. The change in expression of death receptor along with FADD was measured after treatment with TRAIL, QC and their combination in DR5-KD cells. There was no change in the expression of DR5 observed, but a little increased expression of DR4 and FADD were noted in TRAIL, QC and their combination treatment. The histogram of Figure 5B showed the cell cycle regulation and apoptosis in DR5-KD cells after the treatment. It was noted that the percentages of apoptosis and the population of S-phase arrest of the cells were lower in comparison to parental (DR5 positive, see Figure 3D) cells (Figure 5B). Approximately, less than 28% S-phase arrest was noted in DR5-KD cells in comparison to parental MCF-10A-Tr cells after exposure to 2.5 μM QC in TRAIL pre-treated cells (compare Figures 3D *vs.* 5B).

To confirm the contribution of cell death mediated by DR5, the apoptosis in DR5-KD cells was determined by annexin V-FITC/PI dual staining method. There was no significant increase in apoptosis noticed in DR5-KD cells after combination treatment (Figure 5C). Approximately, 40% less cell death was noticed in DR5-KD cells in comparison to parental MCF-10A-Tr cells (compare Figures 3F *vs.* 5C), confirming a significant DR5 contribution for causing the apoptosis in cells. The un-alteration of ROS and NO production after treatment with TRAIL, QC and their combination in DR5-KD cells further confirmed that DR5 mediates its action through mitochondrial pathway (Figure 5D and 5E).

To confirm further the involvement of DR5 in QC mediated apoptosis, we have over-expressed the WT DR5 in DR5-KD cells. These cells were treated with QC, TRAIL and in combination of TRAIL+QC. The expressions of DR5, DR4 and FADD were restored to a level similar to parent cells (Figure S13A). Further, the CASPASE 3 expression was enhanced and apoptotic nuclei were observed in these cells (Figure S13).

### QC specifically binds to DR5 and activates the apoptosis cascade

**Figure 6:**
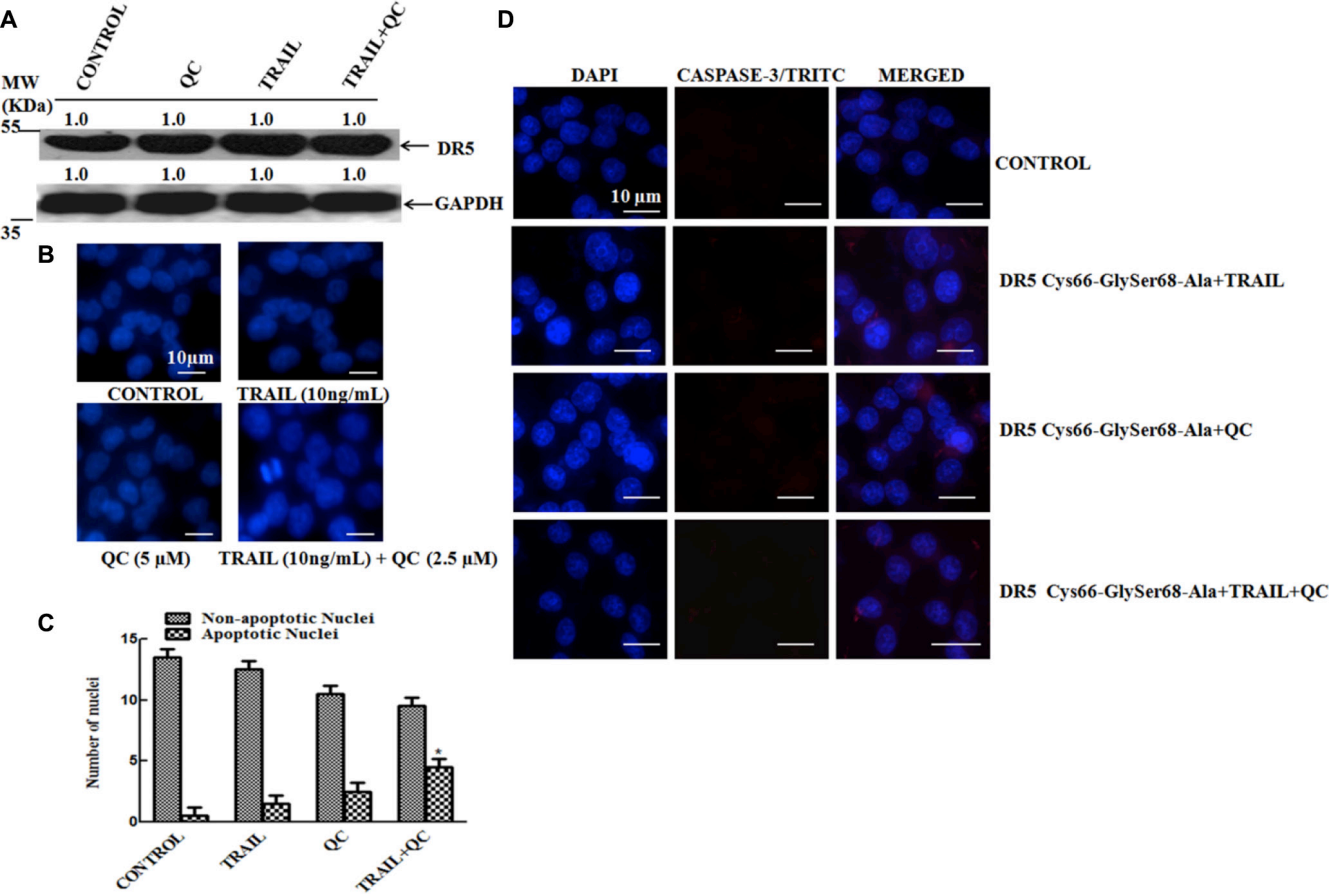
QC specifically interacts in the ectodomain of DR5 and enhances its apoptotic activity Mut DR5 (DR5 Cys66-Gly Ser68-Ala) was transiently over-expressed in DR5-KD MCF-10A-Tr cells and treated with TRAIL, QC and their combination. (**A**) Measurement of DR5 expression by western blotting. (**B**) Measurement of apoptosis by DAPI staining. (**C**) Graph representing number of apoptotic and non-apoptotic nuclei from Figure 6B. (**D**) Measurement of CASPASE 3 activity by immunofluorescence. Images were taken at 40× magnification in Olympus microscope. Images shown are representative of three different experiments (10 μm).

*In silico* data revealed that QC strongly interacts with the ectodomain of DR5 at Cys66 and Ser68 with hydrogen bond and the interaction was abolished after Cys66-Gly and Ser68-Ala mutations (Figure 1A). The results were further confirmed by *in vitro* mutational studies. At first the single amino acid mutations were performed *i.e.* Cys66-Gly and Ser68-Ala and finally a double mutant *i.e.* Cys66-Gly Ser 68-Ala was prepared by side directed mutagenesis. The mutated c-DNA (single DR5 Cys-Gly or DR5 Ser-Ala or double DR5 Cys66-Gly Ser68-Ala) were transiently transfected in DR5-KD cells and then treated with the agents. Cells were harvested post-treatment and western blot was performed in the whole cell lysate to check the expression of DR5. It was noted that after treatment with TRAIL+QC in a single mutated c-DNA (*i.e.* Cys66-Gly or Ser68-Ala) transfected cells minor increase in DR5 expression was noted (data not shown). There was no significant change in the DR5 expression after treatment with any compounds (Figure 6A) in the double mutant (Cys66-Gly, Ser68-Ala) transfected cells. To confirm the interactions further, we have measured the apoptosis in the TRAIL+QC treated cells by DAPI nuclear staining and CASPASE 3 activation. There was no significant increase of fragmented, bubble shaped and shrunken nuclei was noted. There was also no significant increase of CASPASE 3 activation noted under similar experimental conditions (Figure 6B and 6D). In order to evaluate the protein expression profile for the mutant construct of DR5, the cells were transfected with the mutant DR5 plasmids. The protein expression was checked with the help of western blot using anti-flag antibody as probe, as our mutant construct was PCMV-Flag DR5. No significant alteration was noticed in the DR5 expression, when compared to its wild type counterpart (Figure S14).

### TRAIL+QC increases the expression of DR5 and reduces the tumor size in xenograft mice

**Figure 7:**
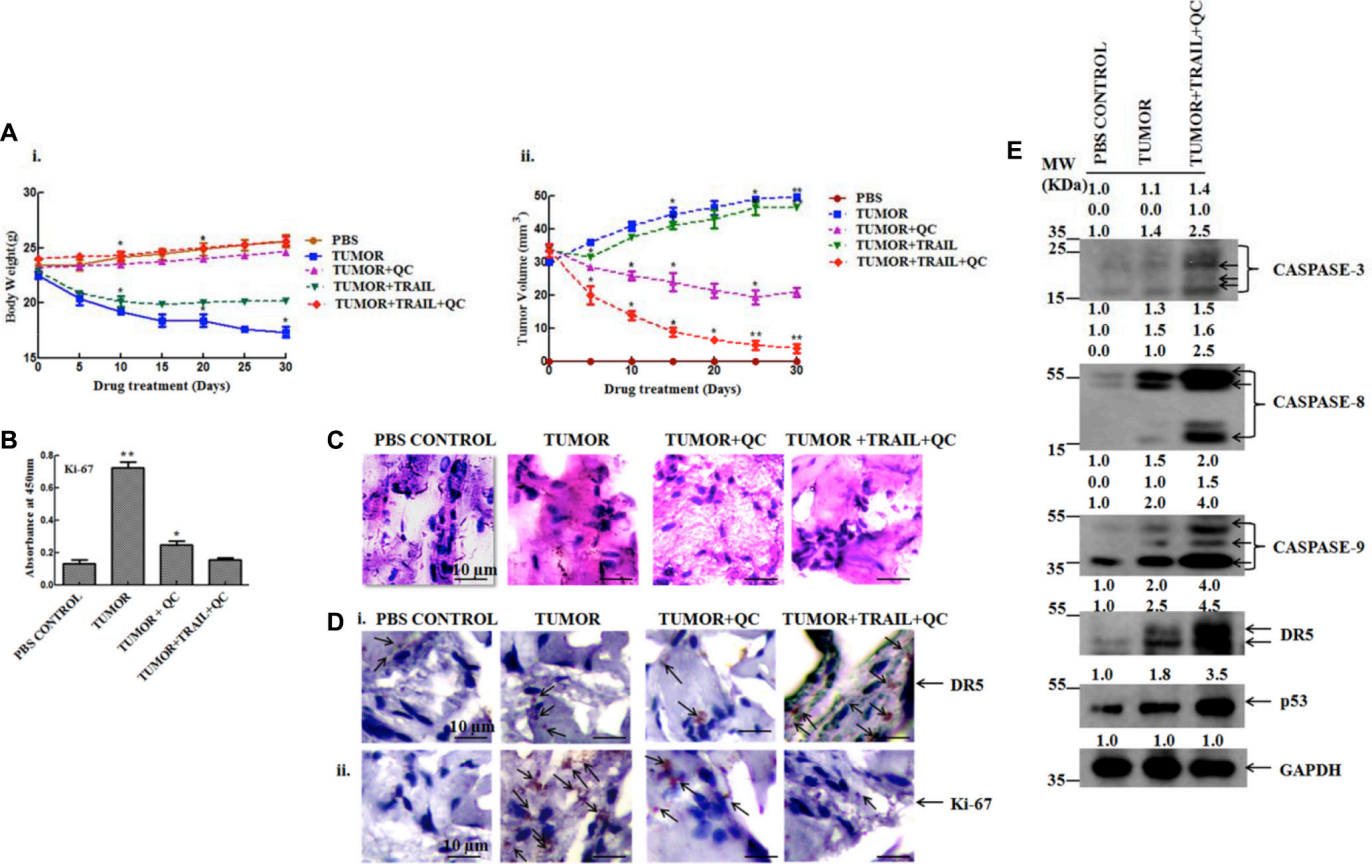
QC caused apoptosis in MCF-10A-Tr cells implanted xenograft mice by induction of DR5 (**A**) i) Change in body weight of mice treated with QC, TRAIL and their combination. ii) Change in tumor volume of QC, TRAIL and TRAIL+QC treated mice. (**B**) Expression of Ki-67. (**C**) H&E staining of the mice breast fat pad tissue section (scale bar 10 μm). (**D**) IHC i) DR5 ii) Ki-67 (scale bar 10 μm; arrows indicate DR5/Ki-67 expression). (**E**) Expression of CASPASE (S), DR5 and p53 in tumor lysate. Images shown are representative of three different experiments. Data are the mean ± SD of three independent experiments.

The effect of TRAIL+QC was evaluated in xenograft mice as per the protocol mentioned in methods and material. Figure 7A.i and 7A.ii showed the increase in body weight and decrease in tumor size upon treatment with QC, TRAIL and their combination. We observed an increase in body weight and decrease in tumor volume up to thirty days of treatment with QC, TRAIL and their combination. Figure 7B showed decrease in the expression of the proliferation marker Ki-67 after treatment in comparison to untreated mice. The protein expressions of the representative apoptosis markers (PARP cleavage, Cyt-c release) in whole tumor lysate clearly indicated the TRAIL+QC treated mice caused apoptosis. Further, the Hematoxylin and Eosin (H&E) staining results demonstrated small and orderly arranged nuclei in treated mice in comparison to untreated one. Untreated mice tissue showed large distorted and irregular arrangement of nuclei (Figure 7C). To further check the status of DR5 and Ki-67 expression in treated xenograft mice tissue, an immunohistochemistry (IHC) was carried out. An enhanced DR5 expression and reduced Ki-67 expression in TRAIL+QC treated tissue was noted in comparison to untreated one (Figure 7D.i and 7D.ii). Measuring the expression of representative apoptosis markers in TRAIL+QC treated tumor lysates showed an increased expression of p53, and CASPASES (3, 8, and 9) in comparison to phosphate buffered saline (PBS) or untreated sample (Figure 7E). In agreement with the cell based data, an enhanced DR5 expression was noted in QC and TRAIL+QC treated mice, as compared to untreated sample (Figure 7E and Figure S15). Thus, all together these data suggests that administration of TRAIL+QC in xenograft mice leads to apoptosis by enhancing the expression of DR5, CASPASE(S) and p53.

## DISCUSSION

The recombinant human TRAIL and several DR5 agonistic antibodies are being explored for their anti-cancer efficacy. Their application in anti-cancer therapy is limited due to their short half-life and development of resistance in cancer patients [35]. Many studies indicated that the use of therapeutic agents (such as oxaliplatin, 5-FU, CPT-11, etc.) and phytochemicals (such as resveratrol, curcumin, etc.) have gained popularity for chemoprevention and cancer treatment [33, 36]. Till date, the application of chemotherapeutic agents is not sufficiently explored for the treatment of TRAIL resistant patients. Quinacrine is an anti-cancer drug that targets a number of cellular pathways for exerting its therapeutic effect. Jani *et al.* [33] (using colon cancer cell lines) and Wang *et al.* [11] (hepatocellular carcinoma cell lines) have showed that QC and TRAIL combination synergistically increased the cellular toxicity. The detailed biochemical mechanism for synergistic action of QC and TRAIL and more importantly, their effect on other cancer cell lines need a thorough exploration. A systematic study on the role of QC in DR5 mediated apoptosis is presented in this work, using *in silico*, *in vitro* (cell based) and *in vivo* (xenograft mice model system) studies.

TRAIL is a natural ligand of DR5 receptor that binds to the ectodomain of DR5. It is capable of efficiently inducing the formation of active receptor trimer, but its ability to promote DR5 clustering is not significant. Contrary to this, Fab fragment of AMG 655 antibody is bivalent in nature and can contribute positively to enhance the TRAIL-DR5 interaction. Graves *et al.* observed an enhanced cellular apoptosis upon the co-administration of TRAIL and AMG 655 [34]. They also observed that TRAIL and AMG 655 are incapable of significantly promoting the cellular apoptosis, individually. These observations support the synergistic action of AMG 655 and TRAIL. Similar observations were made in case of QC and TRAIL treatment by Wang *et al.* [11] and Jani *et al.* [33], which were re-established in this study also. From these observations, it can be hypothesised that QC and AMG 655 are involved in analogous mode of action. With this assumption, the molecular modeling studies were performed.

To delineate the underlying mechanism of the TRAIL and QC mediated apoptosis, the *in silico* studies were performed, and the results obtained from *in silico* studies were further validated using cell based model system. The structure of the ternary complex, QC-TRAIL-DR5 generated from the molecular docking studies was compared with the binary complex TRAIL-DR5 and ternary complex Fab-TRAIL-DR5. The TRAIL-DR5 affinity was found to be higher in the presence of Fab or QC as compared to that in the binary complex. Further, the hydrogen bonds between TRAIL and DR5 were more in number with a higher stability (indicated by a higher occupancy) in the ternary complexes. The analysis of 3D complexes revealed that the Fab fragment binds mainly to the DR5, whereas QC acts as glue that holds the TRAIL and DR5 together. The reduced volume of interfacial cavities in the presence of Fab and QC further establishes that TRAIL and DR5 are brought closer (Figure S8), increasing their interactions. This is probably resulting in enhanced cellular death signaling and apoptosis, observed *in vitro*. A comparison of the binding energies of QC-TRAIL-DR5 complex and Fab-TRAIL-DR5 complex showed that QC is a better DR5 agonist as compared to the AMG 655 Fab. The 3D structure analysis shows that QC acts as a bridge between DR5 and TRAIL, strengthening the TRAIL-DR5 complex and probably, this factor is responsible for the observed enhancement in cellular apoptosis. An increase in the binding affinity of TRAIL and DR5 was not observed when the residues CysB66 and SerB68 were mutated to Gly and Ala, respectively. This indicates the importance of these two residues in mediating the cytotoxic potential of QC. The mutation of these important residues (Mut DR5 Cys66-GlySer68-Ala) by site directed mutagenesis led to un-alteration of DR5 expression after treatment with TRAIL+QC. This confirms that TRAIL+QC interact with DR5 at the site predicted from *in silico* studies. From the MD results of QC-TRAIL-DR4 complex, no additional gain was noticed (neither in the form of energy nor in the form of proximity between TRAIL and DR4). Absence of any significant increase in the TRAIL-DR4 interaction in the presence of QC or Fab (Figure S9) can be inferred to the specificity of QC for DR5.

MCF-10A-Tr cells are developed from MCF-10A by continuous and repeated exposure of low dose of cigarette smoke extract produced from commercially available Indian cigarette. This cell line is known as a good model to study the specific effects of death receptor agonistic agents, such as QC and TRAIL [37]. The suitability of these cell lines is attributed to their higher aggressiveness and surface expression [37] of DR5 than MCF-7 and other breast cancer cells (Figure 2C). Using different cancer cell lines (breast, colon, and kidney) including newly developed cigarette smoke induced breast cancer cells (MCF-10A-Tr); it was shown that QC dose dependently increased the expression of death receptor DR4 and DR5, with a higher effect on DR5 expression (Figure 2). This subsequently led to enhanced apoptosis. Interestingly, this alteration of DR4/DR5 expression and enhanced apoptosis was not noticed in normal breast epithelial cells MCF-10A after treatment with QC (Figure 2). Thus, data suggests that QC increased the DR5 expression and caused apoptosis in cancer cells without affecting normal cells. In agreement with cell based assays, a dramatic increase in DR5 expression was also noted in QC treated xenograft mice (Figure 7).

The apoptosis caused by QC is not directly proportional to DR5 expressions, showing only 15% apoptosis, in spite of more than 75% over expression of DR5 in MCF-10A-Tr cells (Figure 2). These results indicate towards the involvement of some other factors in mediating the QC and DR5 interaction. To address this hypothesis, cells were incubated with a very specific DR5 agonistic ligand TRAIL, with subsequent addition of QC. Simultaneous treatment of MCF-10A-Tr cells with TRAIL (10 ng/mL) and QC (2.5 μM) led to an increase in apoptosis from 15% (QC treatment, Figure 2F) to 44% (TRAIL+QC treatment, Figure 3D). The exposure of TRAIL pre-treated cells with QC exhibited a much higher apoptosis at a comparatively low dose of QC. Thus, it can be concluded that TRAIL synergized the apoptotic action of QC in cancer cells.

The evaluation of molecular signaling pathways involved in QC mediated apoptosis led to some interesting results, which were in agreement with the literature reports regarding the effect of QC on mitochondria. Changchien *et al.* showed that QC causes apoptosis by damaging the mitochondria in the K562 leukemic cell line [38]. In agreement with above observations, our data also showed that TRAIL+QC treatment led to increased ROS production, BAX activation, CASPASE activation and changes the mitochondrial membrane potential (Figure S12). This suggests the activation of the mitochondrial intrinsic pathway resulting into apoptosis. Attenuation of increased ROS and RNS production by their specific inhibitor NAC and L-NAME further confirmed the involvement of mitochondrial damage after QC exposure in TRAIL pre-treated cells.

**Figure 8:**
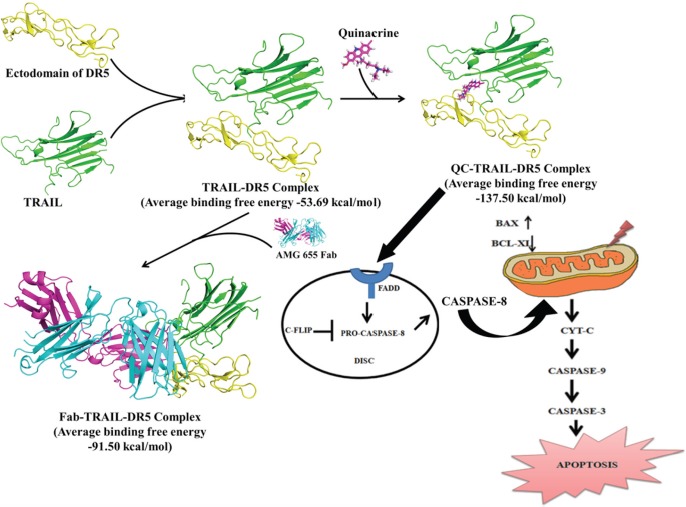
Schematic diagram of TRAIL mediated anti-cancer action of QC by DR5 through mitochondrial intrinsic cascade

In conclusion, the molecular modeling studies together with the *in vitro* studies performed in this work showed that the increase in the cellular toxicity due to QC treatment is mediated through the modulation of DR5 death receptor signaling. TRAIL aggregates into a trimer and binds to the ectodomain of DR5 leading to cellular death. The presence of QC significantly enhances the binding affinity of DR5 with TRAIL by holding the two macromolecules closer. Based on the current results and the known molecular biology, the apoptosis process due to DR5 signaling pathway can be summarised as given in Figure 8. Exposure of QC to TRAIL pre-treated cells helps in effective receptor clustering of TRAIL with DR5 ectodomain. This interaction leads to the recruitment of FADD, an adaptor molecule. FADD then recruits pro-caspase 8 which undergoes autocatalysis to form active CASPASE 8. FADD together with CASPASE 8 forms the DISC and leads to mitochondrial intrinsic pathways. Thus, the co-administration of QC and TRAIL exhibits higher cytotoxicity as compared to their individual administration. This work for the first time reports the atomic level mechanistic details for the DR5 mediated synergistic action of QC and TRAIL in the various cell lines, including carcinogen induced cancer cells.

## MATERIALS AND METHODS

### Small molecular and macromolecular structure preparation for molecular modeling studies

The small molecule considered in this study is QC. The 3D structure of QC was prepared using the Ligand build utility of Maestro9.3 package [39]. The p*K*_a_ and vertical ionization energy calculations were carried out using MarvinView [40] and Gaussian03 [41] software (see supporting information for details) (Figure S1, Table S1, Table S2). The LigPrep module of Maestro9.3 was used to prepare the ligand for molecular modeling studies. The various ionization states possible at physiological pH were generated using Epik ionizer tool available in LigPrep of Maestro package.

The crystal structure of DR5 ectodomain in complex with TRAIL is available in RCSB/PDB [42] as a ternary complex of TRAIL-DR5 ectodomain with Fab fragment from a DR5 agonist antibody, AMG 655 (Fab-TRAIL-DR5) at a resolution of 3.3 Å (PDB ID: 4N90) [34]. For the molecular modeling studies, the representative system was identified by evaluating the binding energies associated with TRAIL-DR5 complexation and finally, one third of the entire crystal structure was selected for the molecular modeling studies. The used structure contained DR5 (chain R), TRAIL (chain A) and Fab (chain E and chain D). The Protein Preparation Wizard of Maestro9.3 software [39] was used to remove water, add the missing hydrogens, assign right bond orders and optimize the orientations of hydroxy group (in Ser, Thr and Tyr), amino group (in Asn and Gln), etc. to the macromolecular systems under consideration. The ionization states of amino acids were optimized with the help of Protassign utility at pH 7.0. Finally, using the impref utility, restrained minimization was carried out (cut-off RMSD of 0.30 Å). Using this, two different macromolecular structures were prepared—one for the binary complex TRAIL-DR5 and the other for ternary complex Fab-TRAIL-DR5. A binary complex of TRAIL-DR5 was created using the prepared Fab-TRAIL-DR5 complex, by removing the coordinates of antibody fragment from the complex structure. This binary complex was used for the molecular docking studies.

### Receptor interaction grid generation and molecular docking

The molecular docking studies of QC in DR5 have been carried out for the first time and there is no information available for the possible binding site of QC in DR5. Considering the mechanistic similarity between AMG 655 (referred as Fab here onwards) and QC, the centroid of Fab interacting residues (ThrA129, ArgA130, SerA145, MetA260, ArgB65, CysB66, GluB70, ValB71, GluB72 and GluB87 where chain A is the TRAIL and chain B is DR5 ectodomain) was used to create the Receptor Interaction grid (center: 36.23, −45.17, 19.88). Finally, molecular docking was performed in Standard Precision (SP) mode, generating 20 poses. The final selection of the best pose was based on the ionization state of QC, molecular docking score and molecular recognition interactions with DR5. The ternary complex of QC, TRAIL and DR5 ectodomain (QC-TRAIL-DR5) was generated from molecular docking and was used for the further molecular dynamics studies. In order to demonstrate the importance of identified binding site residues in mediating the action of QC, *in silico* mutational studies were performed. For this purpose, the two mutations were inserted i.e. Cys66Gly and Ser68Ala in DR5 ectodomain of the ternary complex QC-TRAIL-DR5, using Accelrys Discovery Studio 2.5 [43]. The ternary complex generated after mutation was subjected to molecular dynamics (MD) simulations.

### MD simulations

After analyzing the 3D crystal structures, it was noted that the trimer of the TRAIL interacts with three molecules of DR5. Each DR5 molecule binds at the interface of two TRAIL molecules. Therefore, the binding energy calculations were performed with the help of crystal structures (using the PDB IDs 1D4V and 4N90). For this purpose, 3D structures of two complexes were prepared *i.e.* TRAIL-DR5 (chain A-chain R) complex and TRAIL-DR5 (chain C-chain R) complex. In this process, the interaction between TRAIL belonging to chain A and DR5 were found to be altered due to the presence of Fab in 4N90 (as compared to 1D4V, where Fab is absent) (explained in the result section). After a systematic evaluation, the binary system containing TRAIL (from chain A) and DR5 was concluded to be sufficient for further molecular modeling studies.

Using the identified representative complex of TRAIL-DR5, four systems *i.e.* the TRAIL-DR5 binary complex, Fab-TRAIL-DR5 ternary complex, QC-TRAIL-DR5 ternary complex and QC-TRAIL-Mutated-DR5 ternary complex were chosen to perform MD simulations using AMBER12 package [44]. After generating the input files for molecular dynamics simulation, (i) an initial minimization, (ii) heating and (iii) equilibration were performed (see supporting information for details). Finally, production run for 20 ns was performed under NPT ensemble (cut-off distance of 12 Å for calculating the non-bonded interactions). The relative binding energy for DR5 and TRAIL were calculated over the 20 ns production run using Molecular Mechanics-Generalized Born Surface Area (MM-GBSA) method [45]. Similar studies were performed for the TRAIL-DR4 interaction analysis, for which the co-ordinates of TRAIL, Fab and QC were copied from the corresponding DR5 complexes (Figure S2 and S3) (see supporting information for details).

### Cell lines and reagents

MCF-7, MCF-10A-Tr, MDA-MB-231, BT-20, ZR-75-1 (breast cancer), HCT-116 (colon cancer), and HEK-293T (kidney), cell lines were maintained in Dulbecco's Modified Eagle's Medium (DMEM) media supplemented with 1% antibiotic (100 U/mL of penicillin and 10 mg/mL streptomycin), 1% L-Glutamine and 10% fetal bovine serum (FBS). MCF-10A, normal breast epithelial cell line was maintained in DMEM/F-12 media supplemented with 10% FBS and additional growth factors like 0.5 μg/mL of hydrocortisone,100 ng/mL of cholera toxin, 10 μg/mL of insulin, 10 ng/mL of epidermal growth factor (EGF) and all cultures were maintained at 37°C humidified atmosphere in 5% CO_2._. Recently, we have established a cigarette smoke condensate (CSC) induced-transformed cell line (MCF-10A-Tr) model that could offer a suitable system to study the mechanism of cellular transformation caused by chemical carcinogens and also could help to investigate the mechanism of potential anti-cancer molecules against aggressive and transformed breast cancer cell types [34]. MCF-10A-Tr cells were developed by repeated and continuous exposure to a single dose of CSC prepared from commercially available Indian cigarette to normal breast epithelial cells, MCF-10A. These cells (MCF-10A-Tr) were capable of anchorage-independent growth, and their anchorage dependent growth and colony forming ability were higher compared to the non-transformed MCF-10A cells. The study explained a higher anchorage dependent growth and colony forming ability in MCF-10A-Tr compared to their parental lines. An increased expression of representative biomarkers of oncogenic transformation (NRP-1, Nectin-4), and anti-apoptotic markers (PI3K, AKT, NFκB) in the MCF-10A-Tr cells were also noticed. Short tandem repeat (STR) profiling of MCF-10A and MCF-10A-Tr cells revealed that transformed cells acquired allelic variation during transformation, and had become genetically distinct. MCF-10A-Tr cells formed solid tumors when implanted into the mammary fat pads of BALB/C mice [37].

Cell culture based chemicals were purchased from Himedia (Mumbai, India) and analytical grade chemicals including QC were purchased from Sigma Chemical Ltd. (St. Louis, MO, USA). TRAIL was procured from PeproTech Inc. DR5 siRNA (h) and scramble siRNA constructs were obtained from Santa Cruz Biotechnology Inc., USA. Anti-DR5 (cat # 3062-3) was procured from MBL International, USA. Anti-BCL-XL (cat # 2764), anti-SURVIVIN (cat # 2803), anti-p53 (cat # 9282), anti-FLIP (cat # 3210), anti-CASPASE 8 (cat # 9746), anti-FADD (cat # 2782), anti-CASPASE 3 (cat # 9662), anti-CASPASE 9 (cat # 9502), anti-BAX (cat # 2772), anti-Cyt C (cat # 4280) and anti-DCR1 (cat # 4756) were procured from Cell Signaling Technology, MA, USA. Anti-GAPDH (cat# sc-25778) and anti-MCL 1 (cat # sc-819) were purchased from Santa Cruz Biotechnology, USA.Anti-DR4 (cat # D3813), anti-FLAG (cat #F3165) and anti-DCR2 (cat #3188) was purchased from Sigma and anti-CIAP2 (cat # 51-9000062) was procured from BD pharmingen, USA. Anti-PARP (cat # AB6079) was purchased from Abcam, Cambridge, UK. In combination treatment, cells were pre-treated with TRAIL (10 ng/mL) for 3 h and then QC (2.5 μM) were added and incubated for 48 h. In this set the individual QC concentration used was 5 μM for 48 h.

### Death receptors expression analysis by FACS

Briefly 1 × 10^6^ cells/well of above mentioned cells were grown in 6 well tissue culture dishes till 70% confluence. Then it was treated with increasing concentrations of QC for 48 h prior to harvest and processed for FACS analysis by the protocol mentioned earlier [46]. Cells were incubated with antibodies in 5% FBS and 0.2% Triton X-100, at 4°C for 2 h. Unbound antibodies were removed by washing twice with phosphate buffered saline (PBS) prior to addition of secondary-rabbit conjugated to tetramethylrhodamine (TRITC) antibody. Finally cells were sorted using flow cytometry (FACS CANTO II, Becton & Dickinson, CA, USA) with an event count of 10,000 cells/sample. Data obtained was analyzed using FACS Diva software.

### Measurement of cytotoxicity using 3-(4,5-Dimethylthiazol-2-yl)-2,5-diphenyltetrazolium bromide (MTT) assay

An MTT assay was carried out to check the anchorage dependent growth of cells according to the protocol referred earlier [29]. Briefly ~8000–10,000 cells of MCF-10A-Tr were grown to 70–80% confluence in triplicates in a 96 well tissue culture plate. Cells were treated with different concentrations of QC, TRAIL and combination of TRAIL and QC (different concentrations of QC were added to cells pre-treated with TRAIL (10 ng/mL for 3 h) and was incubated further for 48 h. Then 0.05% MTT was added and incubated at 37°C to allow the formation of formazan crystals. Then the crystal was solubilised by the addition of DMSO and finally the color intensity was measured spectrophotometrically at 570 nm by using microplate reader (Mithras LB 940, Berthold, Germany). Data obtained was represented as percent viability and IC_50_ was determined.

### Analysis of combined drug effect by isobologram diagram

Synergistic, additive or antagonist drug effects were determined by isobologram analysis [31]. Isobologram plots were drawn by plotting the individual IC_50_ values of the drugs in their respective X-(TRAIL) and Y-axis (QC). The IC_50_ values were obtained from the individual drugs effect of MTT assays. Then, a line was used to join both the data points and the IC_50_ value of the combined drug was spotted on the same plot. In principle, if the spotted point (IC_50_ value of combined drugs) falls on the line then it is considered as additive, whereas if it falls below or above the line, then it is considered as a synergistic or antagonist drug effect, respectively.

### Clonogenic cell survival assay

In order to determine the effects of the compounds on the survivability of the cells clonogenic assay was performed as per the protocol referred earlier [29]. Briefly 500 cells/well of MCF-10A-Tr were seeded in a 12 well plate and incubated for 24 h. Cells were treated with different concentrations of QC, TRAIL and TRAIL+QC (different concentrations of QC was added to 10 ng/mL TRAIL pre-treated cells for 3 h). After 48 h of incubation drug treated media was removed and fresh media was added and the cells were allowed to grow for 5–6 doublings. After formation of colony, the media was removed and stained with crystal violet. The crystal violet stained plate was washed with water and the colonies were counted. The data was calculated and expressed as percent survival. Finally the percent survival was plotted against concentrations of the drug to calculate the LC_50_ (fifty percent cell death in culture).

### Western blot analysis

Briefly 1 × 10^6^ cells were grown and treated as above. Cells were harvested and the whole cell lysates were processed for western blotting as per the protocol mentioned earlier [29]. Each blot is a representative of three independent experiments. The number above the blot represent relative fold change in comparison to control analyzed by densitometer by using Image J software.

### Cell cycle analysis

Briefly 1 × 10^6^ cells were treated as above and processed for FACS analysis as per the protocol referred earlier [29]. Cells were fixed with 70% ethanol and stored in −20°C for overnight. The DNA content was evaluated in a FACS scan flow cytometer after staining the cells with PI (50 μg/mL) containing RNase (0.5 μg/mL) and kept for 15 min at room temperature in dark. DNA content of the cells at different phases of cell cycle was analyzed with an event count of 10,000 cells per sample by using FACS (FACS CANTO II, Becton & Dickinson, CA, USA). Result obtained was analyzed using FACS Diva software.

### 4′,6-Diamidino-2-phenylindole (DAPI) nuclear staining

Nuclear fragmentation and cellular apoptosis was observed by staining the cells with DAPI as per the protocol mentioned earlier [29]. Briefly, 10,000 cells/well of MCF-10A-Tr were grown and treated as above. Cells were washed with PBS fixed with ice chilled acetone: methanol (1:1) and kept in −20°C for 15 min. Cells were stained with DAPI incubated for 15 min in dark and apoptotic nuclei were observed under 40× magnification of fluorescence microscope (Evos Fluorescence Microscope, Thermo Fisher Scientific, MA, USA).

### Apoptosis measurement by Annexin V-FITC/PI dual staining

Approximately 1 × 10^5^ cells of MCF-10A-Tr were grown and treated as above before harvest and experiment was carried out according to the Annexin V-FITC/PI detection kit protocol (Sigma). Cells were sorted using Flow cytometer (Attune NxT Flow Cytometer) at an event count of 10,000 cells/sample. The data obtained was analyzed by using FCS express 5 software.

### Death inducing signaling complex (DISC) analysis

Approximately 1 × 10^6^ MCF-10A-Tr cells were grown and treated as above. The pellet was lysed with RIPA lysis buffer. The lysate was cleared by centrifuging twice at 16,000 g for 10 min at 4°C. The soluble fraction was pre-cleared with 20 μl sepharose-4B bead for 2 h at 4°C and immunoprecipitated with anti-DR5 antibody at 4°C for overnight. Beads were then recovered by centrifugation and washed twice with lysis buffer. Then protein was separated by SDS-PAGE and western blot was carried out to check the expression profile of the specific proteins [47].

### Intracellular reactive oxygen species (ROS) and reactive nitrogen species (RNS) measurement

Briefly, 1 × 10^6^ MCF-10A-Tr cells and MCF-10-A-Tr DR5 –KD cells were grown and treated as above. Then the cells were stained with dichloro-dihydro-fluorescein diacetate (DCFH-DA) (10 mM/mL) in fresh media and incubated for 4 h. The cells were then harvested by centrifugation followed by a wash with 1X PBS. The pellet obtained was re-suspended in PBS and ROS production was studied using FACS with an event count of 10,000 cells/sample. The data obtained was analyzed by using FACS Diva Software [48].

For measurement of RNS the drug treated media was taken in a 96 well plate and Griess reagent I (1% sulfonamide in 5% phosphoric acid) and Griess reagent II (0.1% napthylenediamine dichloride) were added [49]. It was incubated for 15 min in dark to allow formation of a pink colour which was then spectrophotometrically analyzed at 560 nm. The values obtained were plotted on graph to determine the nitric oxide (NO) production.

### Silencing of DR5 in MCF-10A-Tr cells

DR5 gene was silenced by using DR5siRNA (h) construct as per the protocol mentioned earlier [31, 36, 50]. Briefly, MCF-10A-Tr (1 × 10^5^ cells/well) cells were grown and transfected with 0.25 μg DR5siRNA (h) and scramble siRNA by lipofectamine 2000 as per the protocol mentioned in the user's manual. After 8 h of transfection cells were supplemented with media containing serum and treated with QC, TRAIL and TRAIL+QC for 48 h. After completion of treatment cells were harvested and processed for different experiments.

### Mutational study

Specific forward primer and reverse primer with a Cys66 mutated to Gly66 and Ser68 mutated to Ala68 of DR5 was synthesized and site directed mutagenesis was performed by using site directed mutagenesis kit as per the user's manual (Invitrogen, CA, USA). Target oligonucleotide sequence required for synthesis of primer was obtained from the cDNA sequence of wild type DR5 (accession # CR541898.1). Wild type DR5 was used as template. Forward and reverse primer for Cys66-Gly mutated sequence is:

5′CATATTTGCAGGAGATGCCATCTCTACCG TCTTCTG 3′ (Reverse primer).

The forward and reverse primer for Ser68-Ala mutated sequence is:

5′GTAGAGATTGCATCGCCTGCAAATATGG AC3′ (Forward primer).

5′GTCCATATTTGCAGGCGATGCAATCTCTAC 3′ (Reverse primer).

The forward and reverse primer for double mutant *i.e.* Cys66-Gly and Ser68-Ala sequence is:

5′CAGAAGACGGTAGAGATGGCATCTCC TGCAAATATG 3′ (Forward primer).

5′CATATTTGCAGGAGATGCCATCTCTA CCGTCTTCTG 3′ (Reverse primer).

Using the above primers and WT DR5 a PCR was run and c-DNA of the mutant DR5 was amplified. It was then transformed into *E. coli* system and purified DNA was isolated. The mutant DR5 (DR5 Cys66-Gly Ser68-Ala) c-DNA (3 μg) was then transiently transfected in DR5 silenced MCF-10A-Tr cells as per the protocol mentioned earlier [50]. Cells were treated as mentioned above and processed for different experiments. Primers were purchased from Integrated DNA Technologies (IDT), Coralville, USA.

### Immunocytochemistry of CASPASE 3

This assay was done to check the expression of CASPASE 3 in DR5-KD MCF-10A-Tr cells after over expressing the cells with DR5-C66G-S68A construct and DR5 WT plasmid. Immunofluorescence of CASPASE 3 was carried out as per the protocol mentioned earlier [51]. Approximately, 20,000 – 30,000 cells were grown on sterile cover slips in a 12 well tissue culture plate. Cells were transiently transfected with mutant DR5 construct by lipofectamine 2000 reagent. The treatment of cells was done as mentioned above. After treatment cells were fixed with acetone: methanol (1:1) and incubated for 20 min in −20°C followed by blocking in 2% BSA with 0.02% triton X-100 in 1X PBS and incubated for 30 min in 37°C. Cells were stained with anti-CASPASE 3 primary antibody and incubated for 2 h in 37°C. Cells were washed with 1× PBS stained with secondary antibody conjugated with TRITC and further incubated for 1 h in 37°C. Nuclei of the cells were counterstained with DAPI and observed under 40× magnification of the fluorescence microscope (Olympus BX61, USA).

### Development of mice xenograft model

*In vivo* experiment with animal model was carried out as per the protocol described earlier [37]. 6 week old female BALB/C mice were maintained in proper light and dark cycle of 12/12 h at School of Biotechnology, KIIT University, Patia, Bhubaneswar, India. Animal ethical Committee (IAEC, School of Biotechnology, KIIT University) has approved all the experimental protocols for animal work. Approximately, 1 × 10^7^ MCF-10A-Tr cells were mixed in 200 μl of freshly prepared sterile PBS and injected into the left mammary fat pad of 3 different groups of mice where each group consisted of 6–8 mice. These mice were monitored every day for tumor formation. When a measurable amount of tumor was marked then 40 mg/kg/day QC, 50 ng TRAIL and 20 mg/kg QC + 50 ng TRAIL was administered orally. PBS was taken as control that was administered likewise. Tumor dimension was measured by means of caliper and volume was calculated by equation ½ (L × W^2^) where Width (W) ≤ Length (L). It was observed that the tumor volume decreased with the increase in days of QC treatment. Body weights of the mice were also measured using weighing balance. After 30 days of treatment the animal was sacrificed and the breast fat tissue was collected. Half of the tissue was used for hematoxylin and eosin (H&E) and immunohistochemistry (IHC). The other half of the tissue sample was homogenized to get the tissue lysate that was further processed and used for western blotting.

### Enzyme linked immunosorbent assay (ELISA)

Expression of the proliferation marker Ki-67 was assayed using ELISA. The experiment was performed as per the protocol mentioned earlier [52]. The pre-coated (Ki-67) plates were incubated with biotin conjugated antibody specific to the Ki-67 protein. Avidin conjugated Horseradish peroxidase (HRP) was added to each well. 3,3′,5,5′-Tetramethylbenzidine (TMB) substrate was added to each well and the enzyme substrate reaction was terminated by adding 0.2 M sulphuric acid. The absorbance was measured at 450 nm.

### H&E staining and immunohistochemistry of xenograft tissue section

H&E and IHC was performed as per the protocol mentioned earlier [37, 52]. The frozen tissues were cut into 5 μm section using Shandon cryotome FSE (Thermo scientific) mounted on SuperFrost^®^ plus slides (Thermo Scientific) at −30°C. For H&E staining slides were rehydrated by immersing in different percentages (100% to 50%) of alcohol. Dried slides were dipped into hematoxylin followed by eosin (H&E) stain. Slides were then dehydrated by immersing in different percentages of alcohol (50% to 100%). Finally slides were incubated in xylene for 2 min and images were captured in bright field microscope using 40× magnification (Leica DM200, USA).

For IHC, fixed slides were blocked in 100 μl blocking solution (5% FBS). It was then immunostained with anti-DR5 and anti-Ki-67 antibody and incubated overnight at 4°C. Slides were then washed with 1× TBST followed by incubation with a HRP conjugated secondary antibody for 30–60 min at room temperature and then developed using 3,3′-Diaminobenzidine (DAB) peroxidase substrate kit (SK-4100, Vector Laboratories, CA, USA). Images were captured at 40× magnification using brightfield microscope (Leica DM2000, USA).

### Statistical significance

Statistical analysis for the comparison of each set of experimental means was performed using GraphPad Prism 5. A two-tailed Student's *t*-test was used where *P < 0.05; **P < 0.001; ***p < 0.0001 was considered to be statistically significant.

## SUPPLEMENTARY MATERIALS


