# Supplementary material for: Quinacrine induces apoptosis in cancer cells by forming a functional bridge between TRAIL-DR5 complex and modulating the mitochondrial intrinsic cascade
Source: Oncotarget. 2016 Aug 17;8(1):248–67. doi: 10.18632/oncotarget.11335 (PMC5352116; doi:10.18632/oncotarget.11335)
Supplement: Supplementary file 1 [file oncotarget-08-248-s001.pdf]

Quinacrine induces apoptosis in cancer cells by forming a functional bridge between TRAIL-DR5 complex and modulating the mitochondrial intrinsic cascade

Supplementary Materials

MATERIALS AND METHODS

Quantum chemical calculations and pKa calculations on quinacrine

In order to identify the possible ionization state of QC at the physiological pH, proton affinity and p*K*a values were calculated by employing the Density Functional Theory (DFT) [1] using Gaussian03 software [2]. The calculations were performed using B3LYP method and 6-31+G(d,p) basis set (Table S1). For the calculation of proton affinity, the QC (B) and the various protonated forms (BH^+) were optimized in the gas phase. The difference in the calculated free energy value of the protonated form (G_{BH^+gas}) and the non-protonated form ($G_{B_{gas}}$) is the desired value of relative energy for two species. The pK_a value and percent concentration of various ionic species of QC at a pH range of 0 to 14 was calculated using MarvinView software [3]. The resulting p*K*a calculations were used to guide the ionic state of QC to be considered for molecular modeling studies (Table S2).

Molecular modeling studies on the TRAIL and DR4 ectodomain complexes

The crystal structure of DR4 ectodomain is not available in the Protein Data Bank (PDB). Therefore, homology modeling technique was adopted to predict and develop the 3-dimensional structure of the same. Sequence of the DR4 ectodomain was retrieved from the UniProtKB database (UniProt accession number: O00220). To identify the homologous proteins and suitable template for the homology modeling, BLAST server was used to perform search against the structural database PDB. The DR5 ectodomain was identified to be the best suitable hit as template (PDB ID: 4N90 [4]; sequence identity: 62%; E-value: $1e^{-41}$). The sequence alignment between template and target sequence was performed using ClustalW server. The MODELLER 9v14 software [5] was used to generate the homology model of the DR4 ectodomain. The generated models were evaluated through SAVES server (http://services.mbi.ucla.edu/SAVES/) by calculating Errat score, Verify 3D score, Ramachandran Plot score, Z-score etc. Loop optimization was further performed using MODELLER 9v14 to improve the structural quality of the models. To generate the 3D structures of the TRAIL-DR4 complex, Fab-TRAIL-DR4 complex and the QC-TRAIL-DR4 complex, coordinates of TRAIL, Fab and QC were taken from the corresponding DR5 complexes. Molecular dynamics studies were performed using the same protocol as that used for DR5 ectodomain.

Molecular dynamics studies

The derivation of partial atomic charges for QC was performed using the semi-empirical (AM1) with bond charge correction (BCC) model [6] available in *antechamber* module of AMBER12. For the preparation of QC and macromolecules, General AMBER Force Field (GAFF) [7] and AMBER ff99SB force field [8] were implemented, respectively. The systems were solvated using TIP3P water, [9] with a solvation box extended to 20 Å in each direction of the solute, forming a cubical box. The initial minimization of the system was performed in three steps. The first and second steps were minimization of the solvent phase with restraints (force constant of 3 kcal/mol/Å^2 and 1 kcal/mol/Å^2 respectively). The third step was an unrestrained minimization. After minimization of the system, gradual heating from 0 to 300K was performed with system restraint of 2 kcal/mol/Å^2 (NVT ensemble) for 50 ps. A three step density equilibration of the protein was carried out under NPT ensemble *i.e.* 50 ps equilibration (restraint 2 kcal/mol/Å^2), followed by another round of 50 ps equilibration (restraint 1 kcal/mol/Å^2) and finally, unrestrained density equilibration for 100 ps. The constant pressure equilibration of 1 ns was performed at 300 K and 1 atm pressure. Finally the production run of 20 ns was performed at 310 K temperature and 1 atmospheric pressure. For entire simulation, the step size was kept 2 fs. A periodic boundary condition was used to perform the production run. During the MD run all bonds containing hydrogen atoms were constrained using SHAKE algorithm [10] Langevin thermostat and barostat were used for temperature and pressure coupling. The non-bonded cut-off was kept at 12 Å, and long-range

electrostatic interactions were treated by the Particle-Mesh Ewald (PME) method [11]. *Ptraj* module of Amber tools, VMD [12] and xmgrace were used for trajectory analysis.

The binding energy calculations were performed on the TRAIL-DR5 binary complex, Fab-TRAIL-DR5 ternary complex and QC-TRAIL-DR5 ternary complex, using MMPBSA method [13]. For the estimation of binding energy in the crystal structures, only a three step minimization was performed and the binding energy calculation was performed on the minimized system. Similar studies were performed for the TRAIL-DR4 ectodomain binary complex, Fab-TRAIL-DR4 ternary complex and QC-TRAIL-DR4 ternary complex, created using the homology modeling with the help of MODELLER 9v14 [5] and copying the coordinates of Fab and QC from the corresponding DR5 complexes.

Mitochondria membrane permeabilisation (MMP) assay

MMP assay was used to check the integrity of the mitochondrial membrane by observing the uptake of mitochondrial staining dye mitotracker red as per the protocol mentioned earlier [14]. Briefly 30,000 cells of MCF-10A-Tr were seeded on a sterile cover slip in a 40 mm tissue culture disc and allowed to reach 70% confluence. Cells were treated as per the treatment regimen mentioned above. After the treatment cells were washed with 1× PBS and fixed with 4% paraformaldehyde for 10 min. Slides were washed in 1× PBS and stained with mitotracker red for 30 min. Nuclei were counterstained with DAPI for 15 min. and cells were visualized under 40× magnification using fluorescence microscope (Olympus BX61, USA).

Over-expression of WT DR5 in DR5-KD MCF-10A-Tr cells

Over-expression of WT DR5 was carried out as per the protocol mentioned earlier [15]. Briefly 1×10^5 cells per well of MCF-10A-Tr–DR 5 KD cells were grown and allowed to attend 70% confluence. 3 μg of PCMV–flag WT DR5 plasmid was transfected by lipofectamine 2000 as per the manufacturer's protocol. After 8 h of transfection, serum containing media was added and treated with QC, TRAIL and TRAIL+QC for 48 h. After completion of treatment, cells were harvested and processed for various experiments.

RESULTS

Homology modeling of DR4 ectodomain

The homology modeling was undertaken for the ectodomain of DR4 receptor. Out of the created 100 models, the best model (on the basis of DOPE score, Ramachandran plot and Errat quality) was used for the loop modeling. The selected model was having a DOPE score of −6393.59. The refined model was satisfactory in terms of Ramachandran plot (99.10% residues in favored region, 0.90% residues in allowed region and 0.00% residues in disallowed region), Errat plot (89.58%) and Verify 3D plot (86.11% of the residues had an averaged 3D-1D score > = 0.20). Errat is a program to analyze the statistics of non-bonded interactions in a protein and is represented as a plot of the error function value (calculated by statistical comparison with highly refined structures) *vs.* position of a 9-residue sliding window. The percentage value is an indicative of the percent residues which have an error value less than 95%. The Verify 3D is a program to analyse the compatibility of tertiary structure with its primary amino acid sequence. The calculations are performed by assigning a structural class to a residue, based on its location and environment (alpha, beta, loop, polar, nonpolar etc) and comparing the results to good structures. The RMSD between the used template and the generated model was calculated to be 0.667 Å (Figure S3). Thus, the resulted model was of acceptable quality to be used for further molecular modeling studies.

System stability analysis

The molecular dynamics simulations were performed on various complexes to evaluate the effect of QC binding on TRAIL-DR5 axis. The systems were stabilized after 10 ns production run, as reflected from the protein backbone RMSD (Figure S5A) and whole protein RMSD (Figure S5B. A significantly higher backbone fluctuation was observed in case of QC-TRAIL-DR5 complex. This is attributed to the conformational changes occurring in DR5 ectodomain to facilitate the interaction with TRAIL. The QC binding to TRAIL-DR5 complex reduces the space between TRAIL and DR5, increasing the binding affinity of TRAIL and DR5 complexation. The protein RMSD (Figure S5B) takes into account the whole protein RMSD, including backbone atoms as well as the side chain atoms. Therefore, the whole protein RMSD is significantly higher than the backbone RMSD. The high RMSD in case of Fab-TRAIL-DR5 complex is due to a higher number of atoms in this complex (Fab contains nearly 300 residues). The residues which exhibited a significantly higher B-factor (Figure S5C) and atomic fluctuation (Figure S5D) were SerB22, SerB24, GluB25, GlyB26, GlyB36, AspB37, GlyB38, ArgB39, AspB40, ThrB105, GlyB106, CysB107, ProB108, ArgB109, GlyB110, MetB111, LysB113, ValB114, GlyB115, AspB116, CysB117, ThrB118, ProB119, GluB124, CysB125, ValB126, HisB127 and LysB128. The topographical location of these residues is shown in Figure S5E. These residues are situated at the two structural ends of the DR5 ectodomain. To facilitate the interaction with TRAIL, the DR5 ectodomain undergoes a conformational change suitable to hold the two

macromolecules closer. This structural change is facilitated by the movement of two ends of DR5 ectodomain towards TRAIL, resulting into the higher B-factor and atomic fluctuation of these residues.

A binary TRAIL-DR5 complex is the representative system

The crystal structure available in RCSB/PDB as PDB ID 4N90 contains three molecules each of DR5, TRAIL and Fab. This is a very huge system to take up for computational studies. Having a symmetric arrangement of three units of TRAIL-DR5-Fab complex, each DR5 molecule interacts with two molecules of TRAIL. Using the molecular modeling studies, the two TRAIL molecules were evaluated for their binding affinities with DR5 in the available crystal structures *i.e.* PDB IDs 1D4V and 4N90. The analysis of crystal structure with PDB ID 1D4V showed that the binding energy for TRAIL-DR5 (chain B-chain A) complexation is −46.39 kcal/mol, whereas that for TRAIL-DR5 (chain E-chain A) complexation −62.98 kcal/mol. This data suggest that the two TRAIL molecules behave differently in complexation with each DR5.

The calculation of binding energy for the TRAIL-DR5 complexation of PDB ID 4N90 (a complex containing DR5, TRAIL and Fab) unveiled some interesting facts about the influence of AMG 655 binding on the DR5 interaction with the two TRAIL molecules. Each Fab (chain E and chain D in 4N90) of AMG 655 actually binds at the interface of TRAIL (chain A in 4N90) and DR5 (Figure S4). The binding energy for Fab-TRAIL-DR5 (TRAIL is from chain A) complex is −83.03 kcal/mol. Thus, Fab binding releases additional 36.64 kcal/mol energy, favouring the TRAIL-DR5 complexation. Such a change was not observed in case of TRAIL (from chain C) and DR5 interactions (−62.98 kcal/mol and −61.22 kcal/mol in PDB IDs 1D4V and 4N90 respectively). Therefore, in rest of the work, a binary complex of TRAIL (chain A) and DR5 (chain R) is considered for the molecular modeling studies.

Effect of QC on cell cycle profile of MCF-7 and MDA-MB-231 cells

The cell cycle profile of MCF-7 and MDA-MB-231 cells upon exposure to varied concentration of QC for 48 h showed an enhanced Sub-G1 phase arrest at higher concentration in comparison to untreated cells. There was a 16% arrest of Sub-G1 population at the highest concentration in MDA-MB-231 cells and a 14% Sub-G1 arrest was marked in MCF-7 cells (Figure S11A and S11B).

Effect of QC, TRAIL and their combination in mitochondrial depolarization mediated apoptosis of MCF-10A-Tr cells

Mitochondrial membrane potential serves to maintain the integrity of mitochondria. Disruption of mitochondrial membrane potential leads to swelling of mitochondria resulting in decreased supply of energy which ultimately leads to cell death. Apoptotic events lead to opening of the mitochondrial transition pore resulting in disruption of $\Delta\psi m$. As shown from Figure S10A mitotracker stained cells treated with QC (5 μM, 48 h), TRAIL (10 ng/ml, 48 h), TRAIL(10 ng/ml, 3 h)+QC (2.5 μM, 48 h) exhibited decreased red fluorescence (polarized state) and increased blue fluorescence (depolarized state) in DR5 positive MCF-10A-Tr cells. In DR5 silenced cell line, reduced red fluorescence was observed in comparison to DR5 positive cell line (Figure S12A and S12B). The intensity of the red fluorescence serves as a measure of the mitochondria membrane potential disruption. Low red fluorescence indicates damaged mitochondria (mitochondrial membrane potential disruption) and high red fluorescence indicates healthy mitochondria (no mitochondria membrane potential disruption) (Figure S12).

Restoration of the apoptotic efficiency after re-expression of WT DR5 in DR5 silenced MCF-10A-Tr cells upon exposure to QC, TRAIL and TRAIL+QC

To check if the apoptotic efficiency is restored upon re-expression of DR5 in DR5 silenced MCF-10A-Tr cells upon the above mentioned treatment; we over-expressed WT DR5 and checked for the expression of DR5, DR4 and FADD along with apoptotic nuclei formation and expression of CASPASE 3. An enhanced expression of DR5, DR4 and FADD was observed in DR5 silenced cells with over expressed WT-DR5, upon exposure to QC, TRAIL and TRAIL+QC (Figure S13A). From DAPI nuclear staining and immunofluorescence of CASPASE 3 under same treatment conditions, we observed an increased apoptotic nuclei and enhanced CASPASE 3 expression (Figure S13B, S13C and S13D). Hence the data here confirms the restoration of the apoptotic efficiency upon re-expression of WT DR5, further confirming the inefficacy of mutDR5 to induce apoptosis even after treatment with QC, TRAIL and TRAIL+QC.

Verification of the protein expression of the mutant constructs

The protein expression of the DR5 mutant constructs was verified by blotting with anti-FLAG antibody. We observed a comparatively low expression of protein in mutant constructs in comparison to pcmv-flag-DR5 (WT) construct and no expression in the vector control (Figure S14).

Apoptosis caused by TRAIL+QC is mediated by up-regulation of DR5

To understand the role of TRAIL and QC on apoptosis in xenograft mice model, the expression pattern of DR5, poly ADP ribose polymerase (PARP) and CYT-C

was checked by western blotting. A 6.4 fold increase in the expression of DR5 was observed in TRAIL + QC treated tissue lysate in comparison to QC (4.2 fold) and TRAIL(1.3 fold).Distinct PARP cleavage was observed in QC and TRAIL + QC treatment in comparison to TRAIL. CYT-C, a marker of mitochondria mediated apoptosis also got enhanced in TRAIL + QC treatment in comparison to QC and TRAIL (Figure S15).This indicated that apoptosis caused by TRAIL + QC involves the up-regulation of DR5 ,PARP and CYT-C which is again in co-relation with cell based data that implies activation of mitochondria mediated intrinsic cascade of apoptosis.

REFERENCES

1. Baboul AG, Curtiss LA, Redfern PC, Raghavachari K. Gaussian-3 theory using density functional geometries and zero-point energies. J. Chem. Phys. 1999; 110:7650–7.
2. Frisch MJ, Trucks GW, Schlegel HB, Scuseria GE, Robb MA, Cheeseman JR Jr, Montgomery JA, Vreven T, Kudin KN, Burant JC, Millam, JM, Iyengar SS, Tomasi J, et al. Gaussian 03, Revision C.02.2004.
3. MarvinView 5.2.3_1; 2009:ChemAxon (http://www.chemaxon.com/marvin).
4. Graves JD, Kordich JJ, Huang, TH, Piasecki J, Bush TL, Sullivan T, Foltz IN,Chang W, Douangpanya H, Dang T. et al. Apo2L/TRAIL and the death receptor 5 agonist antibody AMG 655 cooperate to promote receptor clustering and antitumor activity. Cancer Cell. 2014; 26:177–89.
5. Sali A, Liz P, Feng Y, Herman van V, Karplus. Evaluation of comparative protein modeling by MODELLER. Proteins: Struct Funct Bioinf.1995; 23:318–26.
6. Jakalian A, Bush BL, Jack DB, Bayly CI . Fast, efficient generation of high-quality atomic charges. AM1-BCC model: I. Method, J Comput Chem. 2000; 21:132–46.
7. Wang J, Wolf RM, Caldwell JW, Kollman PA, Case DA. Development and testing of a general amber force field. J Comput Chem. 2004; 25:1157–74.
8. Hornak V, Abel R, Okur A, Strockbine B, Roitberg A, Simmerling C. Comparison of multiple Amber force fields and development of improved protein backbone parameters. Proteins. 2006; 65:712–25.
9. Jorgensen WL, Chandrasekhar J, Madura JD, Impey RW, Klein ML. Comparison of simple potential functions for simulating liquid water. J Chem Phys. 1983; 79:926–35.
10. van Gunsteren WF. Berendsen HJC. Algorithms for macromolecular dynamics and constraint dynamics. Mol Phys. 1977; 34:1311–27.
11. Darden T, York D, Pedersen L. Particle mesh Ewald: An N.log (N) method for Ewald sums in large systems. J Chem Phys. 1993; 98:10089–92.
12. Humphrey W, Dalke A, Schulten K.VMD: visual molecular dynamics. J Mol Graph. 1996; 14:33–38
13. Kollman PA, Massova I, Reyes C, Kuhn B, Huo S, Chong L, Lee M, Lee T, Duan Y, Wang W. Calculating structures and free energies of complex molecules: Combining molecular mechanics and continuum models. Acc Chem Res. 2000; 33:889–97.
14. Satapathy SR, Mohapatra P, Das D, Siddharth S, Kundu CN. The apoptotic effect of plant based nanosilver in colon cancer cells is a p53 dependent process involving ROS and JNK cascade. Pathol Oncol Res. 2015; 21:405–11.
15. Preet R, Mohapatra P, Das D, Satapathy SR, Choudhuri T, Wyatt MD, Kundu CN. Lycopene synergistically enhances quinacrine action to inhibit Wnt-TCF signaling in breast cancer cells through APC. Carcinogenesis. 2013; 34:277–86.

Supplementary Table S1: Vertical ionization energies due to protonation of quinacrine at various nitrogens

Molecule	Energy (in Hartrees)		Vertical ionization energy (in kcal/mol)
	G_{gas}	$GQC_{gas}-G_{gas}$	
A	−1593.78	0.00	000.00
B	−1594.17	0.39	245.81
C	−1594.15	0.38	236.80
D	−1594.16	0.38	239.10
E	−1593.78	−0.39	000.32
F	−1594.16	0.01	242.43

The quantum chemical calculations performed on neutral and protonated quinacrine using DFT method at B3LYP6-31+G(d,p) level (1,2).

Supplementary Table S2: The concentration of each ionic species at various pH conditions

pH	A)	B)	C)	D)	pH	A)	B)	C)	D)
**0**	0.00	0.00	0.00	100	**7.2**	0	0.07	2.32	97.60
**0.2**	0.00	0.00	0.00	100	**7.4**	0	0.11	3.63	96.25
**0.4**	0.00	0.00	0.00	100	**7.6**	0.01	0.18	5.63	94.19
**0.6**	0.00	0.00	0.00	100	**7.8**	0.03	0.27	8.62	91.08
**0.8**	0.00	0.00	0.00	100	**8**	0.06	0.41	12.99	86.54
**1**	0.00	0.00	0.00	100	**8.2**	0.14	0.6	19.07	80.18
**1.2**	0.00	0.00	0.00	100	**8.4**	0.32	0.85	27.05	71.77
**1.4**	0.00	0.00	0.00	100	**8.6**	0.69	1.16	36.7	61.44
**1.6**	0.00	0.00	0.00	100	**8.8**	1.41	1.49	47.22	49.87
**1.8**	0.00	0.00	0.00	100	**9**	2.72	1.81	57.29	38.18
**2**	0.00	0.00	0.00	100	**9.2**	4.92	2.07	65.48	27.53
**2.2**	0.00	0.00	0.00	100	**9.4**	8.41	2.23	70.62	18.74
**2.4**	0.00	0.00	0.00	100	**9.6**	13.60	2.28	72.06	12.06
**2.6**	0.00	0.00	0.00	100	**9.8**	20.83	2.20	69.62	7.35
**2.8**	0.00	0.00	0.00	100	**10**	30.16	2.01	63.60	4.24
**3**	0.00	0.00	0.00	100	**10.2**	41.18	1.73	54.79	2.30
**3.2**	0.00	0.00	0.00	100	**10.4**	52.96	1.40	44.46	1.18
**3.4**	0.00	0.00	0.00	100	**10.6**	64.29	1.08	34.06	0.57
**3.6**	0.00	0.00	0.00	100	**10.8**	74.17	0.78	24.79	0.26
**3.8**	0.00	0.00	0.00	100	**11**	82.04	0.55	17.30	0.12
**4**	0.00	0.00	0.00	100	**11.2**	87.89	0.37	11.69	0.05
**4.2**	0.00	0.00	0.00	100	**11.4**	92.01	0.24	7.72	0.02
**4.4**	0.00	0.00	0.00	100	**11.6**	94.81	0.16	5.02	0.01
**4.6**	0.00	0.00	0.01	99.99	**11.8**	96.66	0.10	3.23	0.00
**4.8**	0.00	0.00	0.01	99.99	**12**	97.87	0.07	2.06	0.00
**5**	0.00	0.00	0.02	99.98	**12.2**	98.65	0.04	1.31	0.00
**5.2**	0.00	0.00	0.02	99.98	**12.4**	99.14	0.03	0.83	0.00
**5.4**	0.00	0.00	0.04	99.96	**12.6**	99.46	0.02	0.53	0.00
**5.6**	0.00	0.00	0.06	99.94	**12.8**	99.66	0.01	0.33	0.00
**5.8**	0.00	0.00	0.09	99.9	**13**	99.78	0.01	0.21	0.00
**6**	0.00	0.00	0.15	99.85	**13.2**	99.86	0.00	0.13	0.00
**6.2**	0.00	0.01	0.24	99.76	**13.4**	99.91	0.00	0.08	0.00
**6.4**	0.00	0.01	0.38	99.61	**13.6**	99.95	0.00	0.05	0.00
**6.6**	0.00	0.02	0.59	99.39	**13.8**	99.97	0.00	0.03	0.00
**6.8**	0.00	0.03	0.94	99.03	**14**	99.98	0.00	0.02	0.00
**7**	0.00	0.05	1.48	98.47					

Supplementary Table S3: Secondary structure content TRAIL-DR5 in various complexes over the last ns trajectory

Macromolecular complex	Helix (%)	Beta Sheets (%)	Random coils (%)
TRAIL-DR5	1.141	46.388	52.471
QC-TRAIL-DR5	1.141	44.867	55.992
Fab-TRAIL-QC	1.141	46.768	51.711

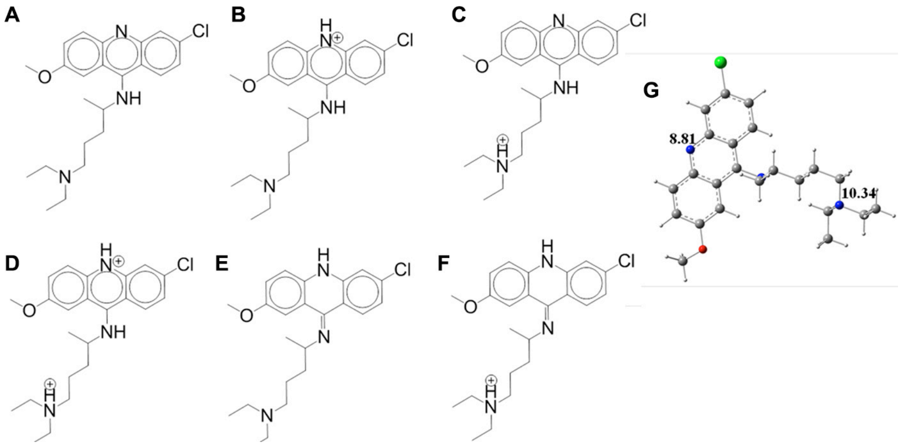

Supplementary Figure S1: Various possible ionization states of QC (A–F) and the predicted p*K*a value of two ionisable nitrogen atoms (G).

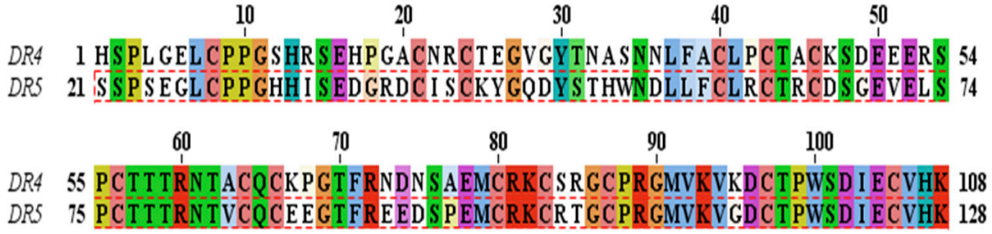

Supplementary Figure S2: Pairwise sequence alignment of DR5 and DR4 ectodomain.

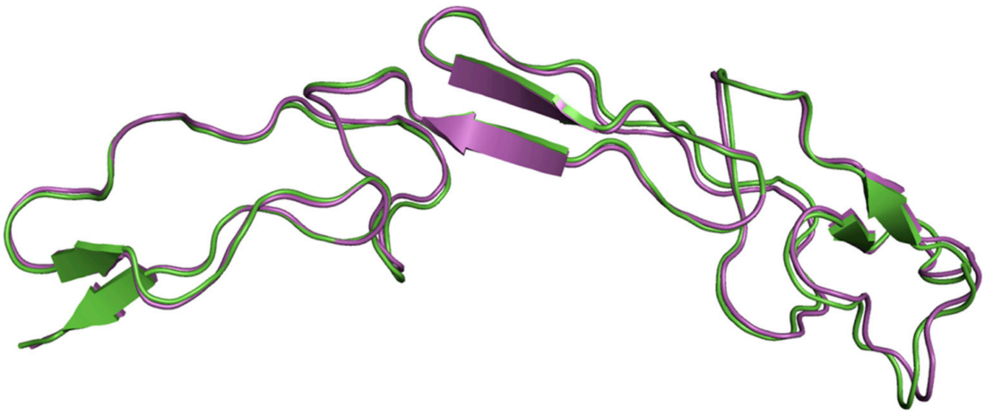

Supplementary Figure S3: Structural alignment of DR4 (magenta) and DR5 (green) ectodomain (RMSD 0.669 Å).

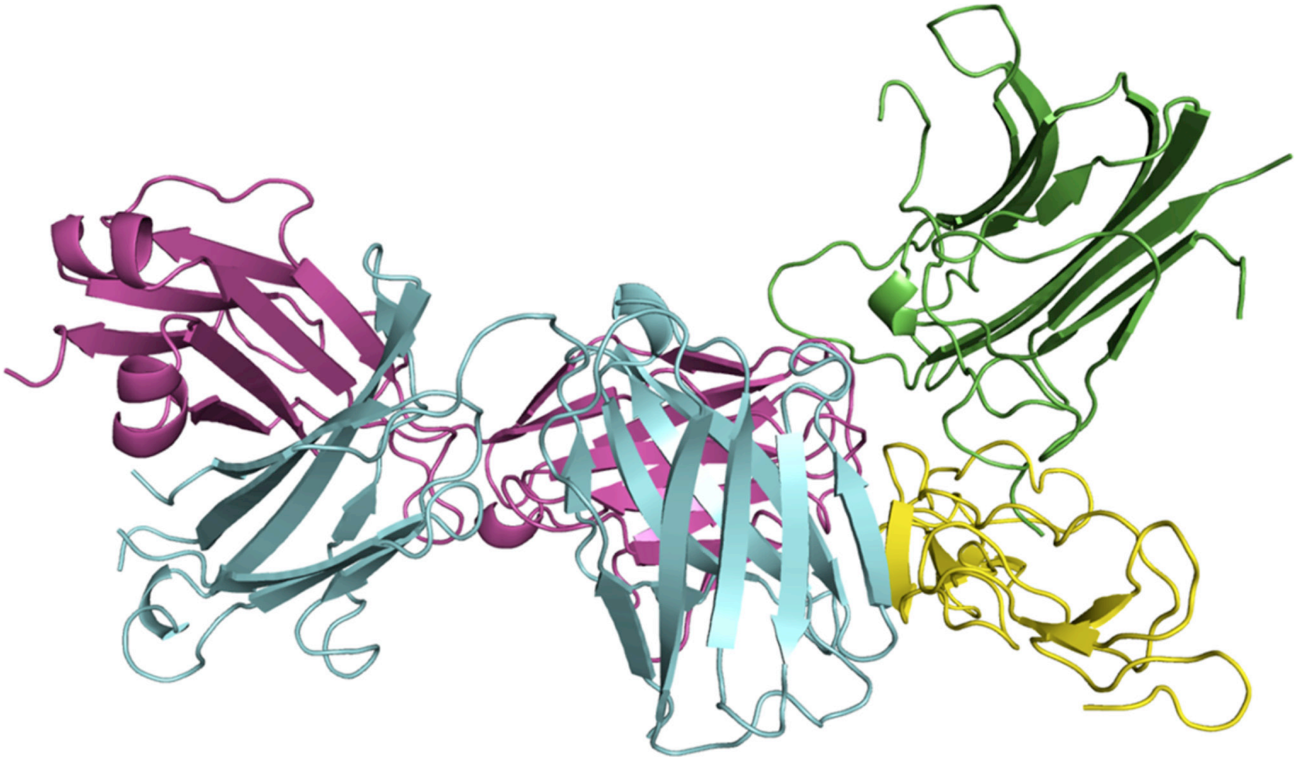

Supplementary Figure S4: The tertiary complex Fab-TRAIL-DR4. DR5 (yellow) is the antigen for the AMG655 antibody (interacts through the Fab domain (cyan and magenta)). The TRAIL (green) shows some minor interactions with the Fab. Fab majorly interacts with the DR5 ectodomain.

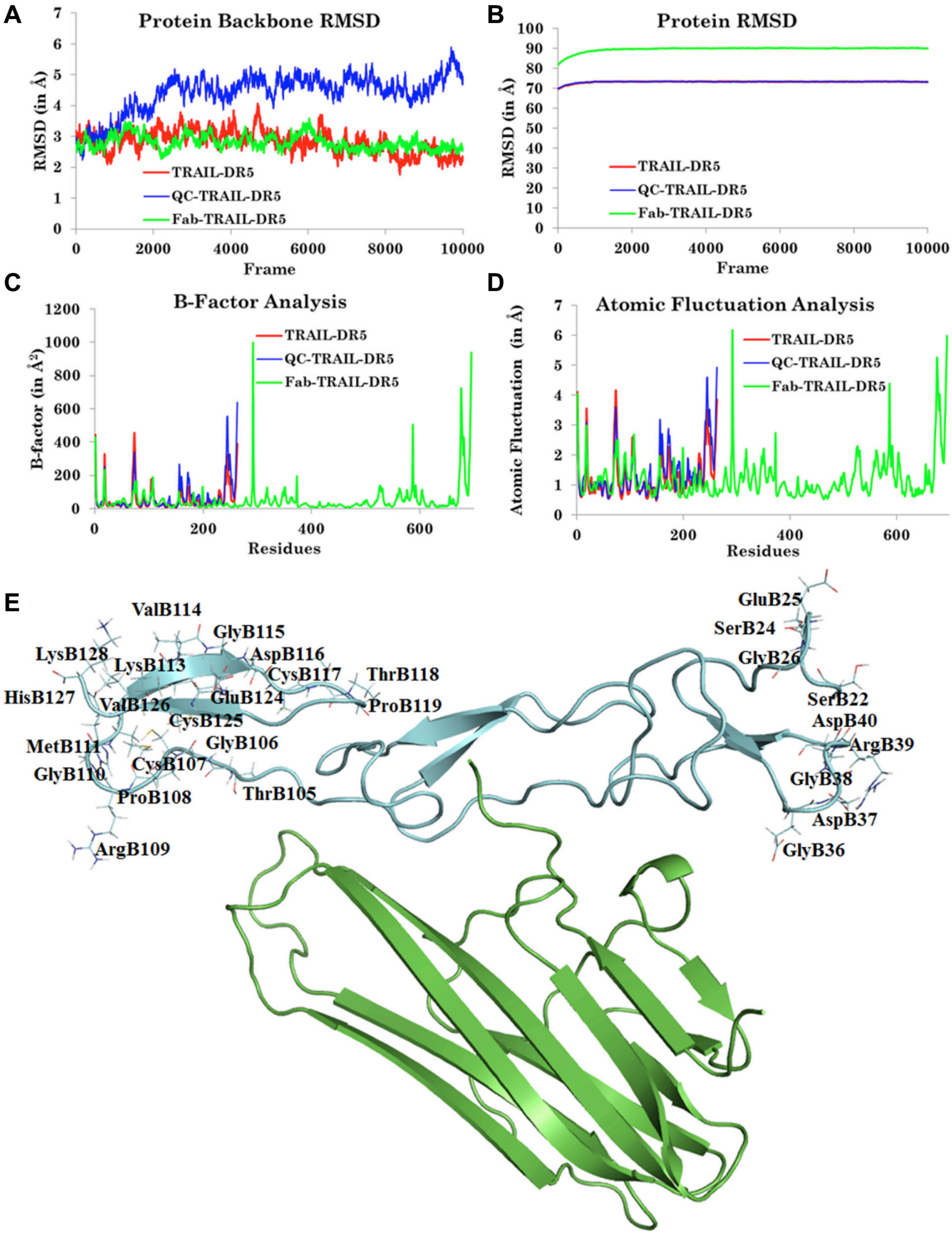

Supplementary Figure S5: System stability analysis after 20 ns molecular dynamics simulation run for the TRAIL-DR5, QC-TRAIL-DR5 and Fab-TRAIL-DR5. (**A**) Protein backbone RMSD. (**B**) Whole protein RMSD. (**C**) B-factor analysis. (**D**) Atomic fluctuation analysis. (**E**) The topographical position of the residues with a higher B-factor in various complexes.

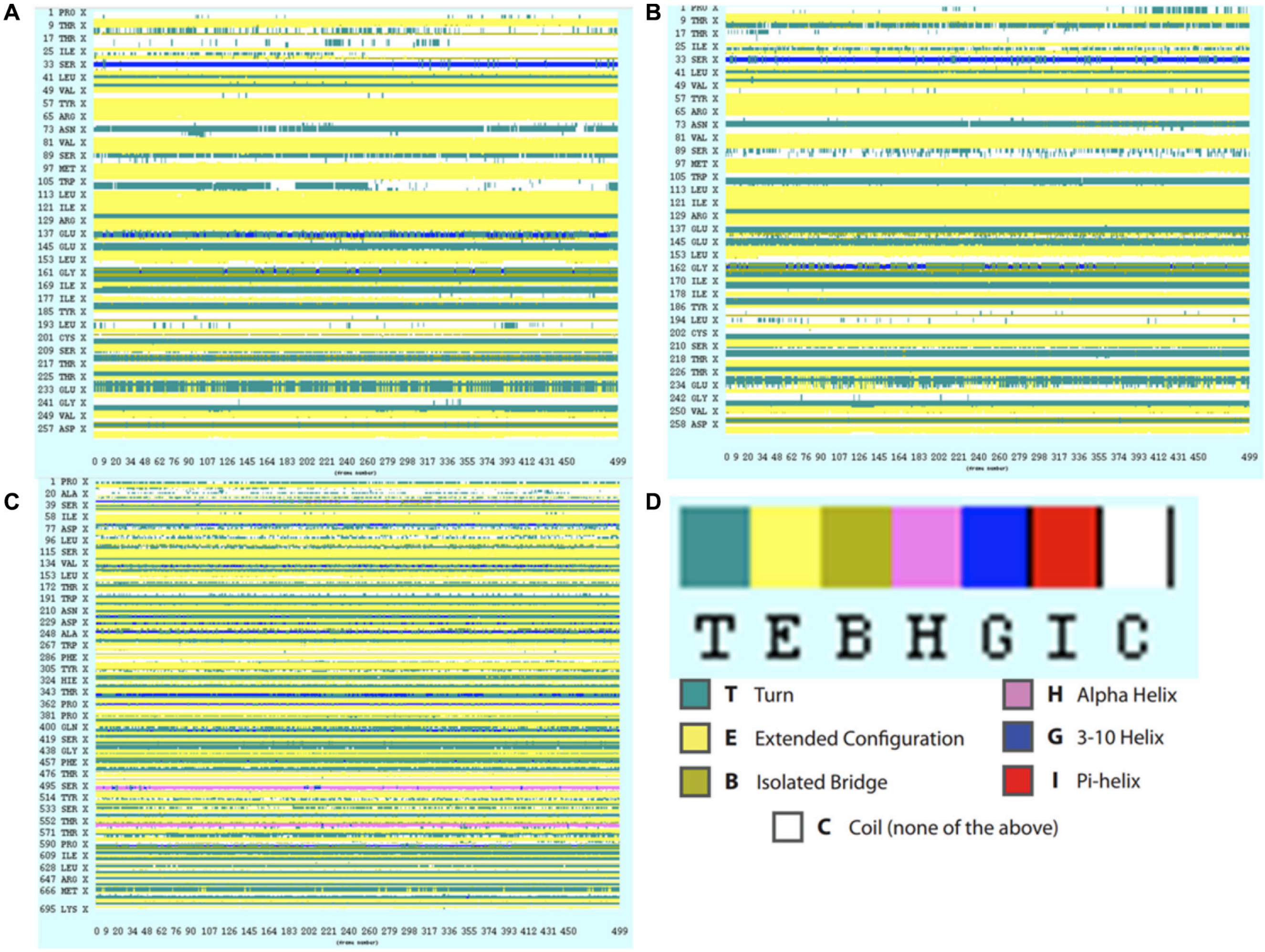

Supplementary Figure S6: Secondary structure content TRAIL-DR5 over the last ns trajectory in (A) TRAIL-DR5. (**B**) QC-TRAIL-DR5. (**C**) QC-TRAIL-DR5. (**D**) Color codes for the secondary structure.

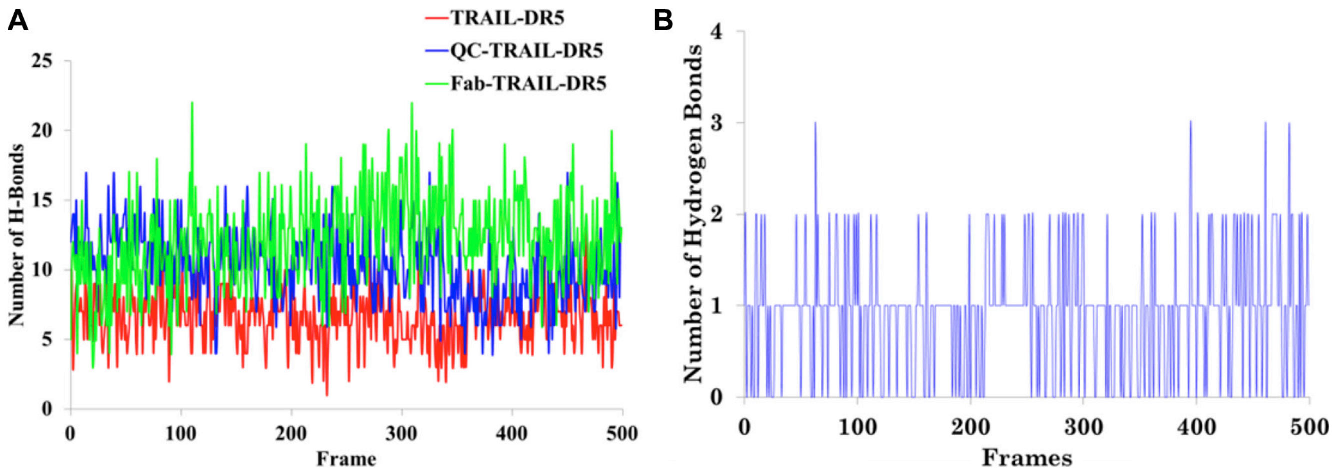

Supplementary Figure S7: Number of hydrogen bonds present over the last ns trajectory (A) between TRAIL and DR5 in the three complexes *i.e.* TRAIL-DR5, Fab-TRAIL-DR5 and QC-TRAIL-DR5. (**B**) between QC, TRAIL and DR5 in the QC-TRAIL-DR5 ternary complex.

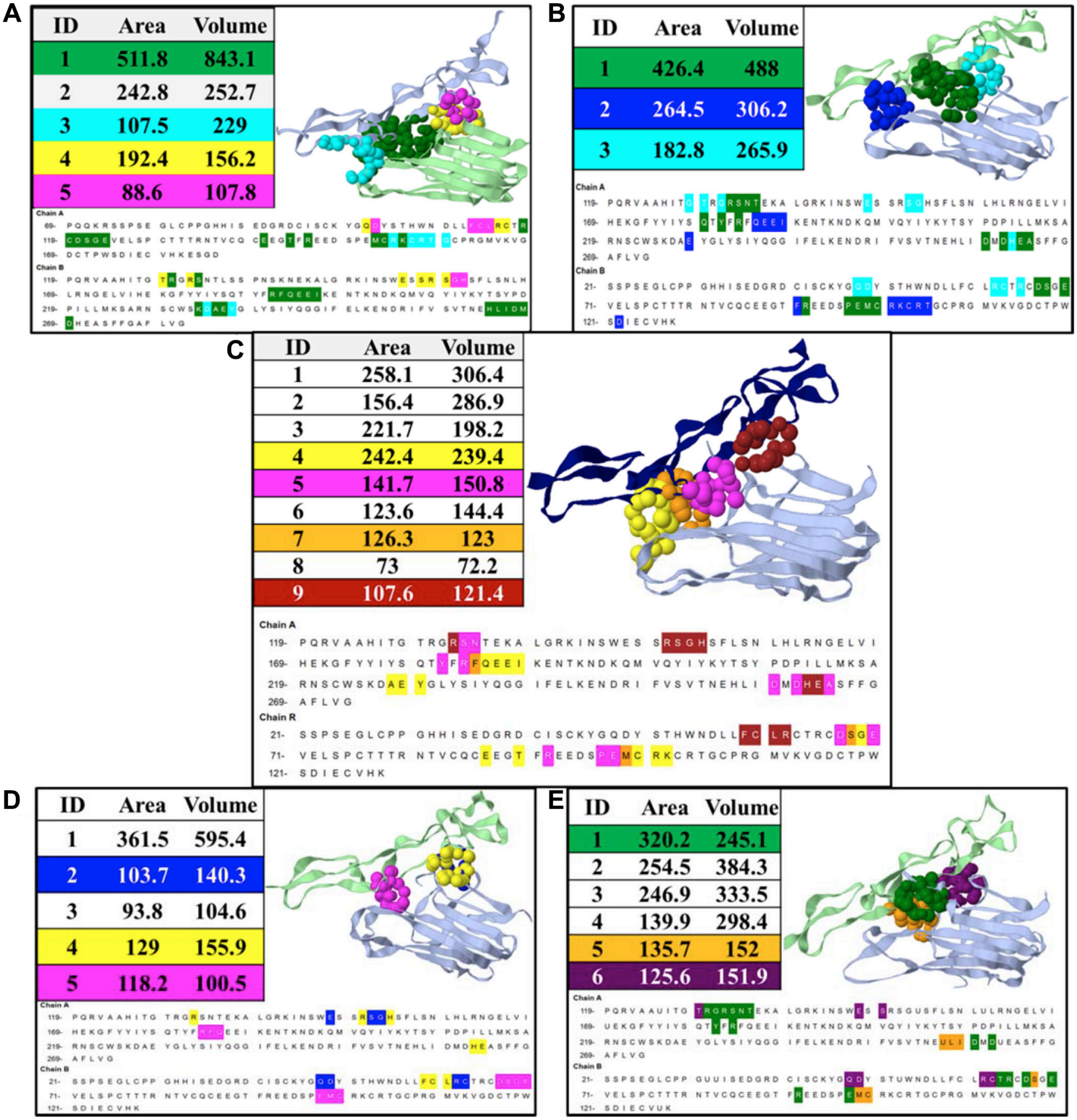

Supplementary Figure S8: Cavity analysis for TRAIL-DR5 in various complexes. (**A**) Crystal structure of TRAIL-DR5 ectodomain (PDB ID: 1D4V; resolution 2.2 Å).[5] (**B**) TRAIL-DR5 after molecular dynamics. (**C**) Crystal structure of ternary complex of Fab-TRAIL-DR5 (PDB ID: 4N90).[4] (**D**) Ternary complex Fab-TRAIL-DR5 after molecular dynamics simulation. (**E**) Ternary complex QC-TRAIL-DR5 after molecular dynamics simulation.

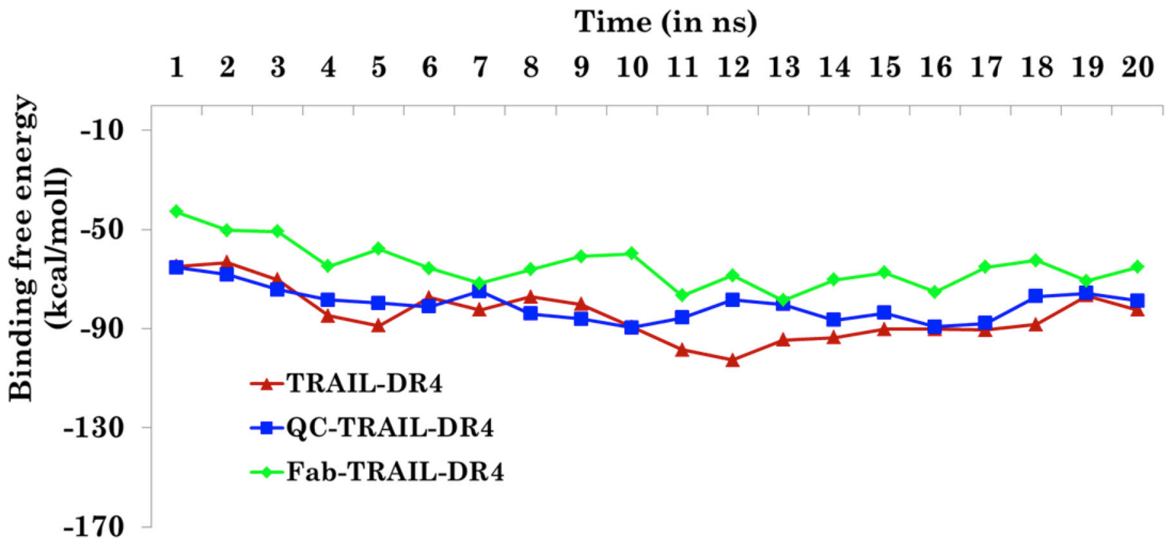

Supplementary Figure S9: The binding energy for three systems containing TRAIL-DR4, QC-TRAIL-DR4 and Fab-TRAIL-DR4.

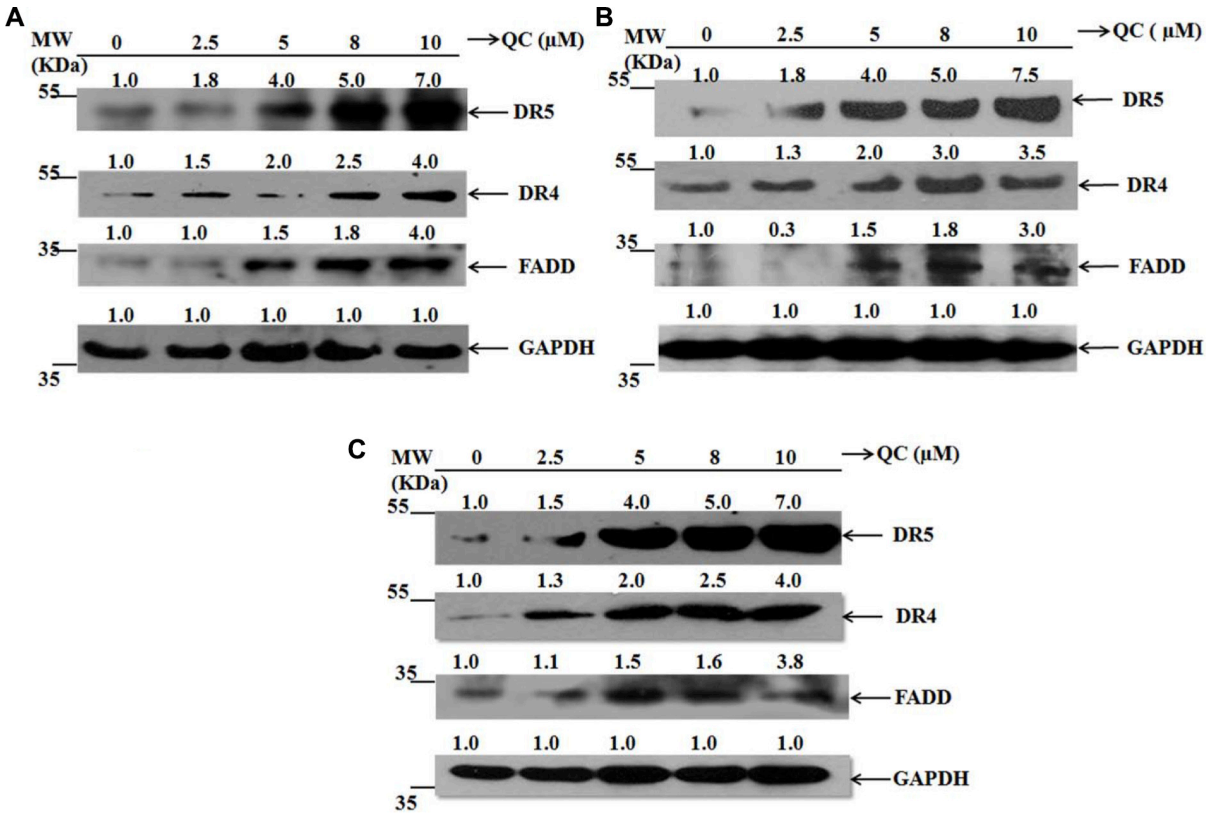

Supplementary Figure S10: Expression of DR5, DR4, FADD in MCF-10A-Tr cells after different concentrations of QC exposure. Figure S10. a is the image from Figure 2A of main manuscript. Given here are three different immunoblots from three different lysates of three different set of experiments.

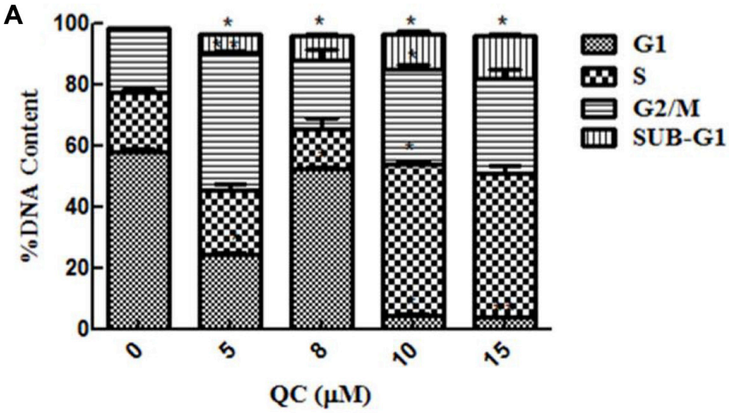

MCF-7

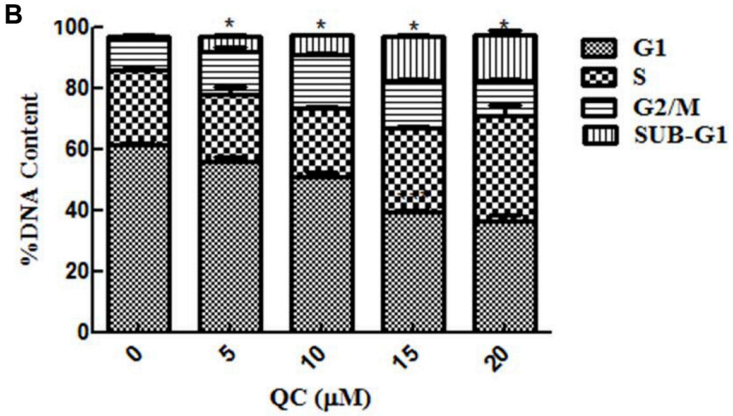

MDA-MB-231

Supplementary Figure S11: QC induced apoptosis in MCF-7 and MDA-MB-231 cells. (**A**) Measurement of apoptosis by FACS analysis in MCF-7 cells. (**B**) Measurement of apoptosis by FACS analysis in MDA-MB-231 cells. Data shown is the representative of three different experiments. Data are the mean ± SD of three different experiments.

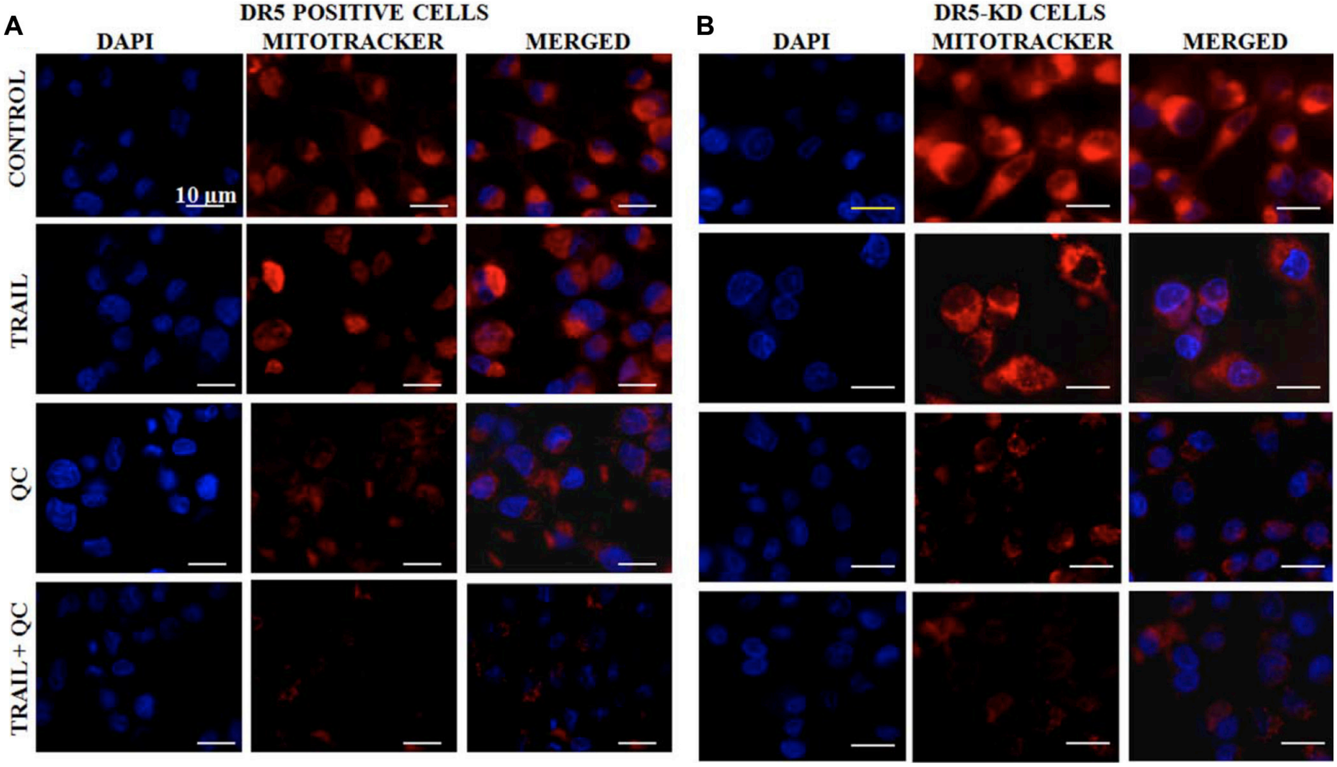

Supplementary Figure S12: **(A)** Fluorescence image of mitochondrial membrane potential (MMP) by mitotracker red in DR5 positive cells. (**B**) Fluorescence image of MMP in DR5-KD cells after mitotracker red staining. All images were taken at 40X in Olympus microscope. Images shown here are representative of three different experiments.

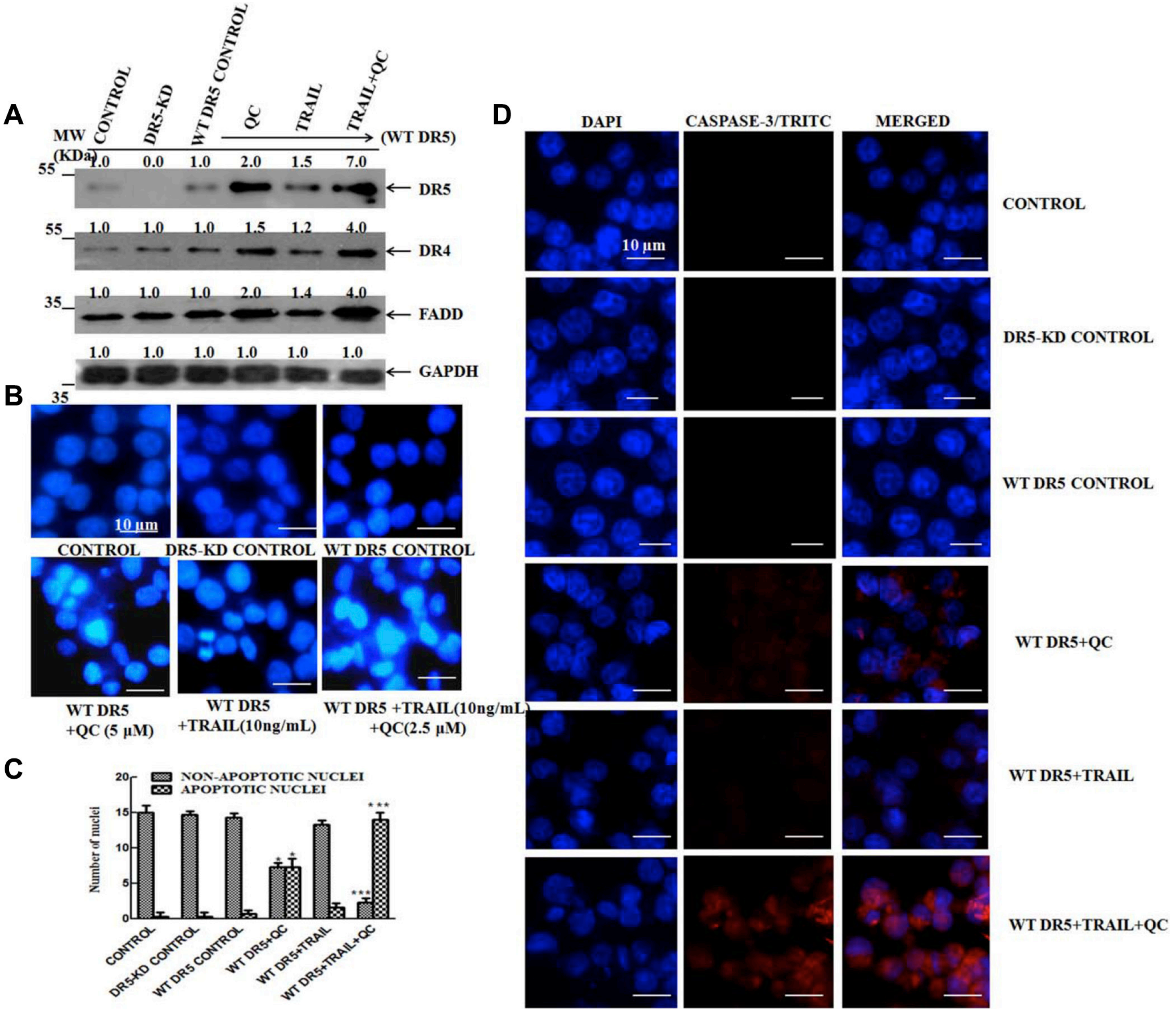

Supplementary Figure S13: Re-expression of WT DR5 restores the apoptotic efficacy in DR5 silenced MCF-10A-Tr cells upon exposure to QC, TRAIL and their combination. (**A**) Immunoblots of DR5, DR4 and FADD. (**B**) Measurement of apoptosis by DAPI nuclear staining. (**C**) Graph representing number of apoptotic and non-apoptotic nuclei from Figure S13B. (**D**). Measurement of CASPASE 3 activity by immunofluorescence. Images were taken at 40× magnification of Olympus microscope. Images shown are representative of three different experiments.

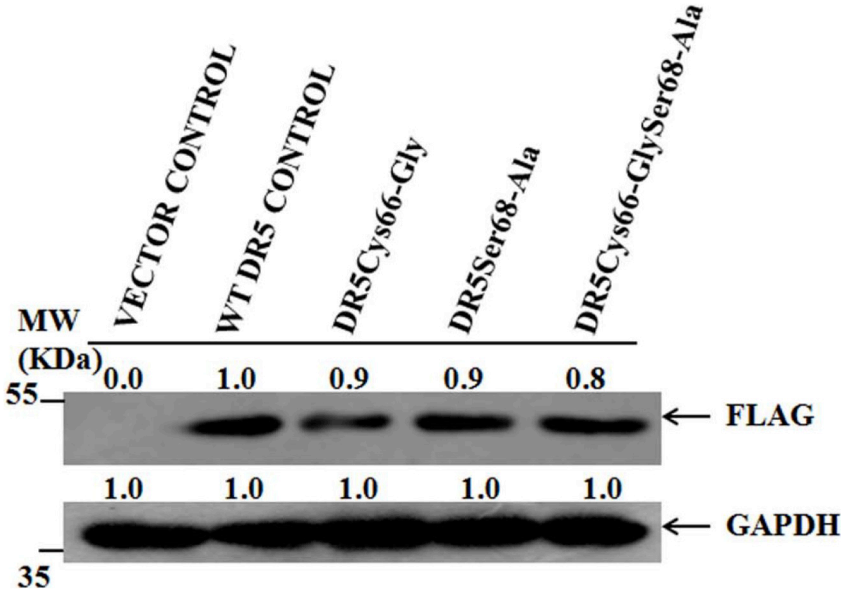

Supplementary Figure S14: Immunoblot showing the protein expression of DR5 after over expression of PCMV FLAG tagged DR5 Cys66-Gly Ser68-Ala construct. The membrane was probed with anti-FLAG antibody. Images shown are representative of three different experiments.

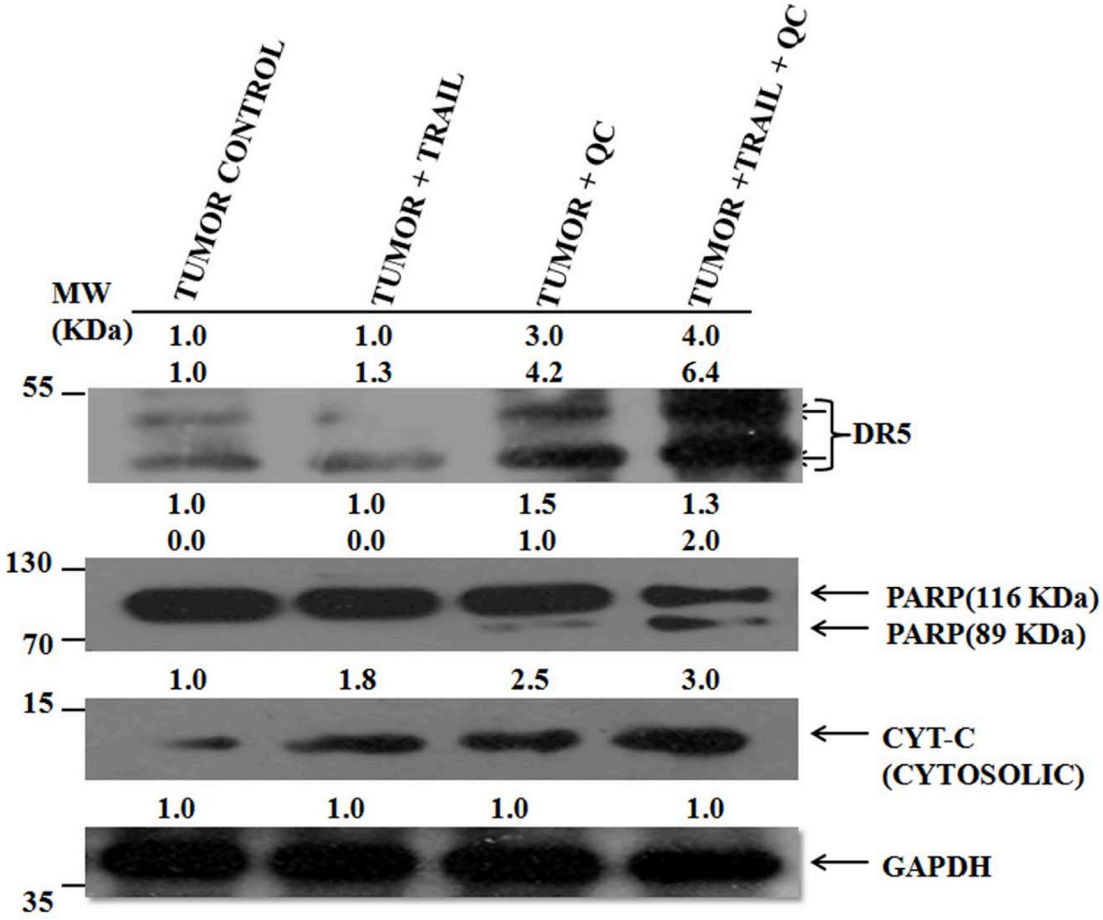

Supplementary Figure S15: Change in expression of DR5, PARP and CYT-C in tissue lysate of xenograft mice after treatment with QC, TRAIL and their combination. The images shown are representative of three different experiments.
